# New 5,6-diphenyl-1,2,4-triazine-hydrazineylidene-phenoxy-1,2,3-triazole-acetamide derivatives as potent synthetic α-glucosidase inhibitors

**DOI:** 10.1039/d5ra06909b

**Published:** 2025-10-15

**Authors:** Nafise Asemanipoor, Shahram Moradi, Mohammad Ali Faramarzi, Maryam Mohammadi-Khanaposhtani, Mohammad Mahdavi

**Affiliations:** a Department of Chemistry,NT.C., Islamic Azad University Tehran Iran shm_moradi@yahoo.com; b Department of Pharmaceutical Biotechnology, Faculty of Pharmacy and Biotechnology Research Center, Tehran University of Medical Sciences Tehran Iran; c Cellular and Molecular Biology Research Center, Health Research Institute, Babol University of Medical Sciences Babol Iran; d Endocrinology and Metabolism Research Center, Endocrinology and Metabolism Clinical Sciences Institute, Tehran University of Medical Sciences Tehran Iran momahdavi@tums.ac.ir

## Abstract

The current work aims to introduce 5,6-diphenyl-1,2,4-triazine-hydrazineylidene-phenoxy-1,2,3-triazole-acetamide derivatives 13a–n as a new class of potent α-glucosidase inhibitors. These compounds were synthesized through well-known and effective chemical reactions in good yields. All title derivatives 13a–n showed high α-glucosidase inhibition in comparison to the standard inhibitor (acarbose). In this regard, the most potent compounds, compounds 13j and 13h, were approximately 6250- and 3947-fold more potent than acarbose, respectively. An *in vitro* kinetics study revealed that compound 13j is an uncompetitive α-glucosidase inhibitor. Molecular docking and molecular dynamics studies on compound 13j revealed highly favorable results, confirming stable binding interactions and robust complex formation of this compound with the enzyme's active site. Furthermore, *in silico* studies indicated that compound 13j possesses favorable and comparable drug-likeness, ADME, and toxicity profiles relative to acarbose, highlighting its potential as a promising lead.

## Abstract 2

The current work aims to introduce 5,6-diphenyl-1,2,4-triazine-hydrazineylidene-phenoxy-1,2,3-triazole-acetamide derivatives 13a–n as a new class of potent α-glucosidase inhibitors.

## Graphical abstract

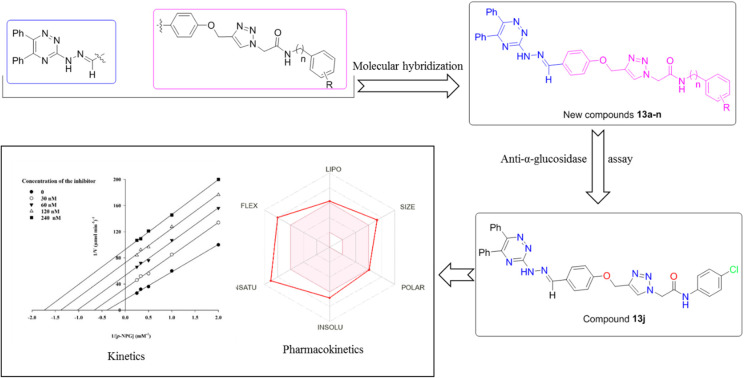

## Introduction

Diabetes mellitus is a metabolic disease in which the body cannot naturally control the level of glucose in the blood.^1^ There are two main types of this disease. In the first type (type 1), the patient suffers from a deficiency of insulin, the most important hormone for controlling blood sugar. In the second type (type 2), which is more prevalent, the body becomes insensitive to the action of insulin.^2^ The increasing prevalence of type 2 diabetes and the increase in deaths from it have made this disease a crisis for the whole world.^3^ Many scientists are trying to find new drugs and treatments for this disease considering the therapeutic mechanisms of this type of diabetes.^4^ Many medications have been introduced for type 2 diabetes, but for long-term use of these medications, their side effects must be tolerable for patients.^5^ Among the therapeutic targets for type 2 diabetes, one of the least risky approaches is to prevent the absorption of carbohydrates.^6^ To this end, the enzymes that break down carbohydrates in the intestine must be inhibited. The most important enzyme in the process mentioned above is α-glucosidase.^7^ In addition to type 2 diabetes, α-glucosidase inhibitors are also used in type 1 diabetes and obesity, but their digestive side effects limit their use.^8^ All of the above points have led us and other research groups to work on designing new inhibitors for this enzyme with greater potency and fewer side effects.^9^ In this path, a valuable tool for us is molecular hybridization of active pharmacophores.^10–12^

N-heterocyclic containing compounds, both natural and synthetic, have always had special importance in medicinal chemistry.^13–16^ These compounds, ranging from amino acids and nucleic acids to complex plant alkaloids, are important building blocks in biology and pharmaceutical sciences.^17^ Some N-heterocycles, such as triazine and triazole, do not occur in nature and are produced synthetically, but they can clearly affect natural processes such as enzyme function.^18,19^ Our target enzyme in this work is α-glucosidase, and various derivatives containing 1,2,4-triazine or 1,2,3-triazole rings with high inhibitory activity against this enzyme were reported.^20,21^ On the other hand, one of the valuable linkers in the design of hybrid molecules as α-glucosidase inhibitors is the Schiff base.^22^

**Fig. 1:**
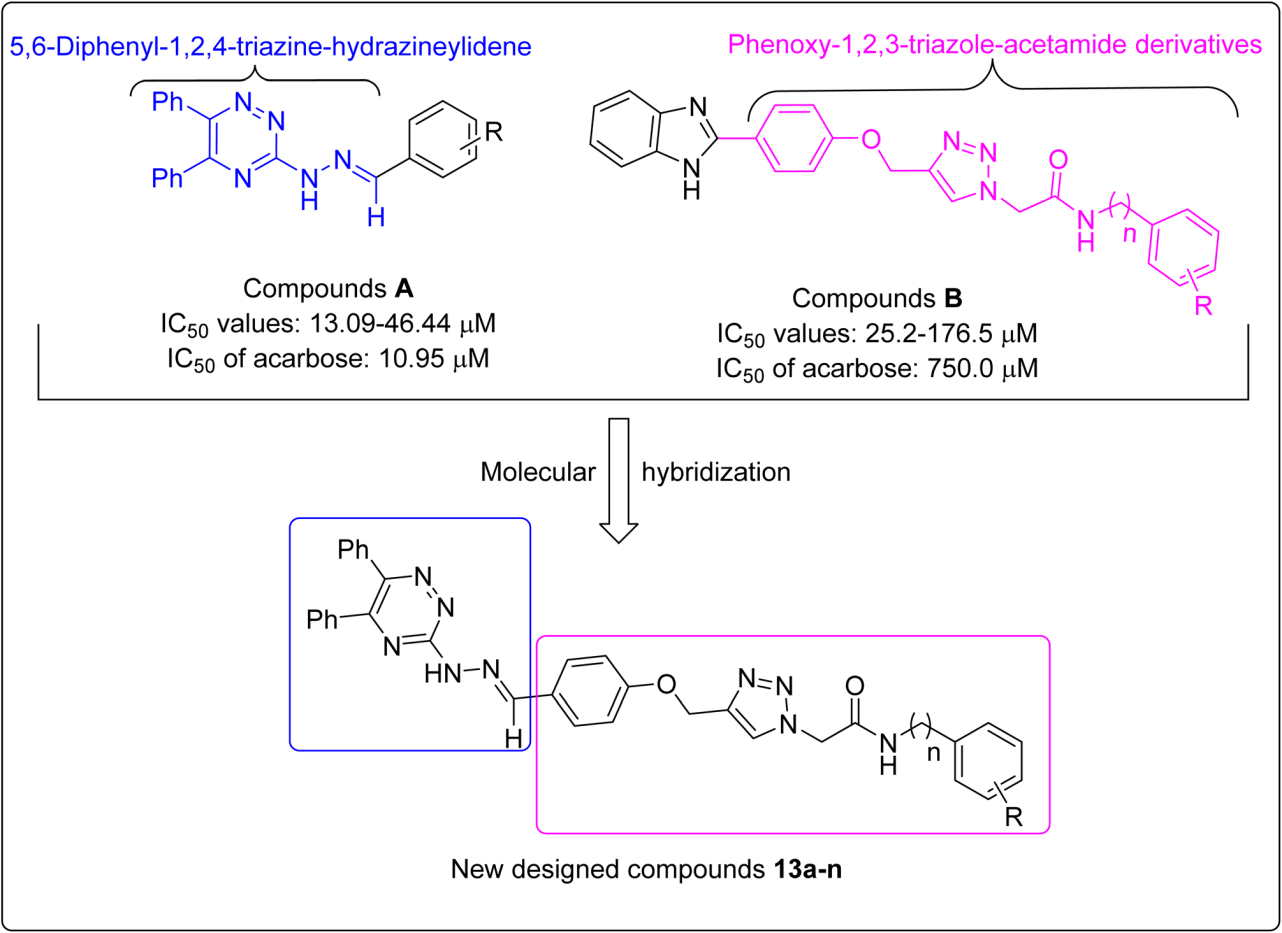
Design strategy for 5,6-diphenyl-1,2,4-triazine-hydrazineylidene-phenoxy-1,2,3-triazole-acetamide derivatives 13a–n.

Our investigations on various α-glucosidase inhibitors containing triazole, triazine, and/or Schiff base structures led to find two series of valuable molecules: 5,6-diphenyl-1,2,4-triazine-hydrazineylidene derivatives A and phenoxy-1,2,3-triazole-acetamides B (Fig. 1).^23,24^ Based on molecular hybridization theory, we attached a 5,6-diphenyl-1,2,4-triazine-hydrazineylidene moiety to phenoxy-1,2,3-triazole-acetamide derivatives to achieve new α-glucosidase inhibitors 13a–n (Fig. 1).^25–30^

## Materials and methods

### Synthesis of 3-hydrazineyl-5,6-diphenyl-1,2,4-triazine (7)

A mixture of benzil (1, 10 mmol) and hydrazinecarbothioamide (2, 10 mmol) in acetic acid (30 mL) was stirred at reflux condition for 6 h. After this time, by adding water, 5,6-diphenyl-1,2,4-triazine-3-thiol (3) was formed and separated by filtration. Compound 3 (10 mmol), ethyl 2-chloroacetate (4, 10 mmol), and K_2_CO_3_ (12.2 mmol) in DMF (30 mL) were stirred at RT for 12 h. To the obtained mixture, water was added, and the resulting precipitates were collected by filtration to give pure ethyl 2-((5,6-diphenyl-1,2,4-triazin-3-yl)thio)acetate (5). The latter compound (compound 5, 10 mmol) and hydrazine (6, 10 mmol) were stirred at 60 °C for 24 h in ethanol (30 mL). As in the previous steps, the workup at this stage also includes adding water and collecting the particles to give pure compound 7.

### General procedure for the synthesis of 1,2,3-triazole derivatives 12a–n

A mixture of 4-hydroxybenzaldehyde (8, 10 mmol), propargyl bromide (9, 12 mmol), and K_2_CO_3_ (12 mmol) in DMF (30 mL) was stirred at room temperature for 8 h. The reaction mixture was then poured into crushed ice, and the resulting precipitate was collected by filtration. The solid was recrystallized from ethanol to afford pure 4-(prop-2-yn-1-yloxy)benzaldehyde (10). Next, a mixture of acetamide derivatives 11a–n (1.1 mmol), sodium azide (0.9 mmol), and NEt_3_ (1.3 mmol) in a water/*t*-BuOH solution (10 mL, 1 : 1 v/v) was stirred at room temperature for 1 h. Subsequently, compound 10 (1 mmol), CuSO_4_ (7 mol%), and sodium ascorbate (14 mol%) were added to the mixture, and the reaction was stirred at room temperature for 24–48 h. After this period, cold water was added, and the resulting products 12a–n were filtered off, washed with water, and purified by recrystallization from ethanol.^21^

### General procedure for the synthesis of 5,6-diphenyl-1,2,4-triazine-hydrazineylidene-phenoxy-1,2,3-triazole-acetamide derivatives 13a–n

In the final step, to produce the target compounds 13a–n in the present work, a mixture of compound 7 (1 mmol), compounds 12a–n (1 mmol), and acetic acid in ethanol was stirred at 60 °C for 10–14 h (checked by TLC). The final compounds were separated by adding water, and the precipitates were collected. The obtained precipitates were recrystallized in ethanol.

### (*E*)-2-(4-((4-((2-(5,6-Diphenyl-1,2,4-triazin-3-yl)hydrazineylidene)methyl)phenoxy)methyl)-1*H*-1,2,3-triazol-1-yl)-*N*-phenylacetamide (13a)

Yellow solid; yield: 85%; MP = 248–249 °C; ^1^H NMR (500 MHz, DMSO-*d*_6_) *δ* 11.33 (s, 1H, NH–N=), 10.48 (s, 1H, NH–C=O), 8.29 (s, 1H, N=CH), 8.26 (s, 1H, triazole), 7.69 (d, *J* = 8.2 Hz, 2H, Ar), 7.59 (d, *J* = 8.0 Hz, 2H, Ar), 7.48 (d, *J* = 7.4 Hz, 2H, Ar), 7.43 (d, *J* = 8.9 Hz, 1H, Ar), 7.39 (d, *J* = 7.4 Hz, 2H, Ar), 7.37–7.36 (m, 2H, Ar), 7.35–7.34 (m, 3H, Ar), 7.33–7.30 (m, 1H, Ar), 7.14 (d, *J* = 8.1 Hz, 2H, Ar), 7.08 (t, *J* = 7.3 Hz, 2H, Ar), 5.37 (s, 2H, CH_2_–C=O), 5.24 (s, 2H, CH_2_–O); ^13^C NMR (126 MHz, DMSO-*d*_6_) *δ* 164.11 (NH–C=O), 159.14 (–N=C–N=), 156.31, 143.92, 142.25, 138.37, 136.15, 135.98 (N=CH), 130.15, 129.38, 128.98, 128.86, 128.32, 128.20, 127.64, 126.34, 123.74, 119.21, 115.03, 61.13 (CH_2_–O), 52.22 (CH_2_–C=O); anal. calcd: C_33_H_27_N_9_O_2_; C, 68.15; H, 4.68; N, 21.67; found: C, 68.32; H, 4.82; N, 21.71.

### (*E*)-2-(4-((4-((2-(5,6-Diphenyl-1,2,4-triazin-3-yl)hydrazineylidene)methyl)phenoxy)methyl)-1*H*-1,2,3-triazol-1-yl)-*N*-(*o*-tolyl)acetamide (13b)

Yellow solid; yield: 85%; MP = 281–282 °C; ^1^H NMR (500 MHz, DMSO-*d*_6_) *δ* 11.84 (s, 1H, NH, NH–N=), 9.80 (s, 1H, NH–C=O), 8.28 (s, 1H, N=CH), 8.24 (s, 1H, triazole), 7.68 (d, *J* = 8.1 Hz, 2H, Ar), 7.48 (d, *J* = 7.5 Hz, 2H, Ar), 7.43 (d, *J* = 7.8 Hz, 2H, Ar), 7.41–7.37 (m, 2H, Ar), 7.39–7.35 (m, 2H, Ar), 7.38–7.34 (m, 3H, Ar), 7.22 (d, *J* = 7.4 Hz, 1H, Ar), 7.17 (d, *J* = 7.3 Hz, 1H, Ar), 7.14 (d, *J* = 9.0 Hz, 2H, Ar), 7.10 (d, *J* = 7.3 Hz, 1H, Ar), 5.41 (s, 2H, CH_2_–C=O), 5.23 (s, 2H, CH_2_–O), 2.23 (s, 3H, CH_3_); ^13^C NMR (126 MHz, DMSO-*d*_6_) *δ* 164.30 (NH–C=O), 159.14 (–N=C–N=), 156.31, 150.59, 143.90, 142.22, 136.15, 135.98 (N=CH), 135.47, 131.55, 130.39, 130.16, 129.37, 128.98, 128.32, 128.21, 127.64, 126.30, 126.02, 125.51, 124.69, 115.03, 61.12 (CH_2_–O), 51.92 (CH_2_–C=O), 17.74 (CH_3_); anal. calcd: C_34_H_29_N_9_O_2_; C, 68.56; H, 4.91; N, 21.16; found: C, 68.75; H, 5.04; N, 21.27.

### (*E*)-2-(4-((4-((2-(5,6-Diphenyl-1,2,4-triazin-3-yl)hydrazineylidene)methyl)phenoxy)methyl)-1*H*-1,2,3-triazol-1-yl)-*N*-(*m*-tolyl)acetamide (13c)

Yellow solid; yield: 85%; MP = 273–274 °C; ^1^H NMR (500 MHz, DMSO-*d*_6_) *δ* 11.85 (s, 1H, NH–N=), 10.42 (s, 1H, NH–C=O), 8.28 (s, 1H, N=CH), 8.26 (s, 1H, triazole), 7.69 (d, *J* = 8.3 Hz, 2H, Ar), 7.48 (d, *J* = 7.5 Hz, 2H, Ar), 7.44 (s, 1H, Ar), 7.43–7.41 (m, 2H, Ar), 7.39 (d, *J* = 8.0 Hz, 2H, Ar), 7.37–7.36 (m, 2H, Ar), 7.36–7.34 (m, 2H, Ar), 7.20 (t, *J* = 7.8 Hz, 2H, Ar), 7.14 (d, *J* = 8.2 Hz, 2H, Ar), 6.90 (d, *J* = 7.5 Hz, 1H, Ar), 5.35 (s, 2H, CH_2_–C=O), 5.23 (s, 2H, CH_2_–O), 2.26 (s, 3H, CH_3_); ^13^C NMR (126 MHz, DMSO-*d*_6_) *δ* 164.03 (NH–C=O), 159.14 (–N=C–N=), 156.31, 150.56, 143.92, 142.23, 138.28, 138.08, 136.15, 135.98 (N=CH), 130.15, 129.37, 128.97, 128.68, 128.31, 128.19, 127.64, 126.31, 124.45, 119.76, 116.40, 115.01, 61.12 (CH_2_–O), 52.23 (CH_2_–C=O), 21.11 (CH_3_); anal. calcd: C_34_H_29_N_9_O_2_; C, 68.56; H, 4.91; N, 21.16; found: C, 68.73; H, 5.06; N, 21.32.

### (*E*)-*N*-(2,4-Dimethylphenyl)-2-(4-((4-((2-(5,6-diphenyl-1,2,4-triazin-3-yl)hydrazineylidene)methyl)phenoxy)methyl)-1*H*-1,2,3-triazol-1-yl)acetamide (13d)

Yellowish white solid; yield: 87%; MP = 290–291 °C; ^1^H NMR (500 MHz, DMSO-*d*_6_) *δ* 11.84 (s, 1H, NH, NH–N=), 9.74 (s, 1H, NH–C=O), 8.27 (s, 1H, N=CH), 8.25 (s, 1H, triazole), 7.68 (d, *J* = 8.2 Hz, 2H, Ar), 7.48 (d, *J* = 7.5 Hz, 2H, Ar), 7.43 (d, *J* = 7.3 Hz, 1H, Ar), 7.42–7.39 (m, 1H), 7.39–7.37 (m, 2H, Ar), 7.37–7.36 (m, 2H, Ar), 7.36–7.34 (m, 2H, Ar), 7.28 (d, *J* = 8.1 Hz, 1H, Ar), 7.13 (d, *J* = 8.2 Hz, 2H, Ar), 7.03 (s, 1H, Ar), 6.96 (d, *J* = 8.0 Hz, 1H, Ar), 5.38 (s, 2H, CH_2_–C=O), 5.23 (s, 2H, CH_2_–O), 2.23 (s, 3H, CH_3_), 2.18 (s, 3H, CH_3_); ^13^C NMR (126 MHz, DMSO-*d*_6_) *δ* 164.23 (NH–C=O), 159.13 (–N=C–N=), 158.58, 156.31, 150.60, 143.90, 142.21, 136.15, 135.98, 134.65, 132.87, 131.51, 130.89 (N=CH), 130.15, 129.37, 128.97, 128.31, 128.20, 127.63, 126.52, 126.28, 124.74, 115.02, 61.12 (CH_2_–O), 51.90 (CH_2_–C=O), 20.41 (CH_3_), 17.66 (CH_3_); anal. calcd: C_35_H_31_N_9_O_2_; C, 68.95; H, 5.13; N, 20.68; found: C, 67.08; H, 5.25; N, 20.71.

### (*E*)-2-(4-((4-((2-(5,6-Diphenyl-1,2,4-triazin-3-yl)hydrazineylidene)methyl)phenoxy)methyl)-1*H*-1,2,3-triazol-1-yl)-*N*-(4-ethylphenyl)acetamide (13e)

Yellow solid; yield: 85%; MP = 252–253 °C; ^1^H NMR (500 MHz, DMSO-*d*_6_) *δ* 11.85 (s, 1H, NH, NH–N=), 10.42 (s, 1H, NH–C=O), 8.28 (s, 1H, N=CH), 8.26 (s, 1H, triazole), 7.69 (d, *J* = 8.2 Hz, 2H, Ar), 7.49 (d, *J* = 8.0 Hz, 2H, Ar), 7.49–7.45 (m, 2H, Ar), 7.43 (d, *J* = 7.3 Hz, 1H, Ar), 7.41–7.38 (m, 1H, Ar), 7.40–7.36 (m, 2H, Ar), 7.38–7.35 (m, 2H, Ar), 7.37–7.32 (m, 2H, Ar), 7.18–7.14 (m, 2H, Ar), 7.16–7.11 (m, 2H, Ar), 5.35 (s, 2H, CH_2_–C=O), 5.23 (s, 2H, CH_2_–O), 2.54 (q, *J* = 7.6 Hz, 2H, CH_2_), 1.14 (t, *J* = 7.6 Hz, 3H, CH_3_); ^13^C NMR (126 MHz, DMSO-*d*_6_) *δ* 163.85 (NH–C=O), 159.14 (–N=C–N=), 158.62, 156.30, 150.57, 143.91, 142.22, 139.17, 136.16, 136.04, 135.98 (N=CH), 130.15, 129.37, 128.97, 128.31, 128.19, 128.03, 127.65, 126.31, 119.31, 115.02, 61.13 (CH_2_–O), 52.19 (CH_2_–C=O), 27.53 (CH_2_), 15.54 (CH_3_); anal. calcd: C_35_H_31_N_9_O_2_; C, 68.95; H, 5.13; N, 20.68; found: C, 67.04; H, 5.28; N, 20.71.

### (*E*)-2-(4-((4-((2-(5,6-Diphenyl-1,2,4-triazin-3-yl)hydrazineylidene)methyl)phenoxy)methyl)-1*H*-1,2,3-triazol-1-yl)-*N*-(4-methoxyphenyl)acetamide (13f)

Brown solid; yield: 85%; MP = 287–289 °C; ^1^H NMR (500 MHz, DMSO-*d*_6_) *δ* 11.83 (s, 1H, NH, NH–N=), 10.34 (s, 1H, NH–C=O), 8.27 (s, 1H, N=CH), 8.24 (s, 1H, triazole), 7.68 (d, *J* = 8.2 Hz, 2H, Ar), 7.50–7.49 (m, 2H, Ar), 7.49–7.47 (m, 3H, Ar), 7.44 (d, *J* = 7.2 Hz, 1H, Ar), 7.39–7.38 (m, 2H, Ar), 7.38–7.37 (m, 2H, Ar), 7.36–7.35 (m, 2H, Ar), 7.14 (d, *J* = 8.2 Hz, 2H, Ar), 6.90 (d, *J* = 8.9 Hz, 2H, Ar), 5.31 (s, 2H, CH_2_–C=O), 5.23 (s, 2H, CH_2_–O), 3.71 (s, 3H, OCH_3_); ^13^C NMR (126 MHz, DMSO-*d*_6_) *δ* 163.56 (NH–C=O), 159.14 (–N=C–N=), 156.31, 155.53, 150.61, 143.90, 143.88, 143.14, 142.20, 136.16, 135.98 (N=CH), 131.45, 130.15, 129.37, 128.97, 128.21, 127.63, 126.29, 120.76, 115.04, 113.99, 61.12 (CH_2_–O), 55.13 (O–CH_3_), 52.12 (CH_2_–C=O); anal. calcd: C_34_H_29_N_9_O_3_; C, 66.76; H, 4.78; N, 20.61; found: C, 66.92; H, 4.81; N, 20.76.

### (*E*)-2-(4-((4-((2-(5,6-Diphenyl-1,2,4-triazin-3-yl)hydrazineylidene)methyl)phenoxy)methyl)-1*H*-1,2,3-triazol-1-yl)-*N*-(2-fluorophenyl)acetamide (13g)

Yellow solid; yield: 85%; MP = 279–280 °C; ^1^H NMR (500 MHz, DMSO-*d*_6_) *δ* 11.84 (s, 1H, NH, NH–N=), 10.33 (s, 1H, NH–C=O), 8.29 (s, 1H, N=CH), 8.26 (s, 1H, triazole), 7.92 (d, *J* = 7.9 Hz, 1H, Ar), 7.69 (d, *J* = 8.2 Hz, 2H, Ar), 7.48 (d, *J* = 7.4 Hz, 2H, Ar), 7.43 (d, *J* = 7.2 Hz, 1H, Ar), 7.40 (d, *J* = 7.9 Hz, 2H, Ar), 7.40–7.36 (m, 1H, Ar), 7.38–7.34 (m, 2H, Ar), 7.37–7.33 (m, 2H, Ar), 7.31–7.25 (m, 1H, Ar), 7.19–7.15 (m, 2H, Ar), 7.14 (d, *J* = 6.8 Hz, 2H, Ar), 5.46 (s, 2H, CH_2_–C=O), 5.24 (s, 2H, CH_2_–O); ^13^C NMR (126 MHz, DMSO-*d*_6_) *δ* 164.75 (NH–C=O), 159.13 (–N=C–N=), 158.59, 156.30, 154.42, 152.47, 150.57, 143.91, 142.28, 136.15, 135.98 (N=CH), 130.15, 129.37, 128.99, 128.32, 128.20, 127.66, 126.36, 125.67, 125.61, 125.47, 125.38, 124.46, 124.44, 123.75, 115.63, 115.48, 115.04, 61.13 (CH_2_–O), 51.99 (CH_2_–C=O); anal. calcd: C_33_H_26_FN_9_O_2_; C, 66.10; H, 4.37; N, 21.02; found: C, 66.22; H, 4.41; N, 21.19.

### (*E*)-*N*-(2-Chlorophenyl)-2-(4-((4-((2-(5,6-diphenyl-1,2,4-triazin-3-yl)hydrazineylidene)methyl)phenoxy)methyl)-1*H*-1,2,3-triazol-1-yl)acetamide (13h)

Yellow solid; yield: 87%; MP = 294–296 °C; ^1^H NMR (500 MHz, DMSO-*d*_6_) *δ* 11.85 (s, 1H, NH, NH–N=), 10.09 (s, 1H, NH–C=O), 8.29 (s, 1H, N=CH), 8.26 (s, 1H, triazole), 7.75 (d, *J* = 8.1 Hz, 1H, Ar), 7.69 (d, *J* = 8.2 Hz, 2H, Ar), 7.51 (d, *J* = 7.9 Hz, 1H, Ar), 7.48 (d, *J* = 7.5 Hz, 2H, Ar), 7.43 (d, *J* = 7.1 Hz, 1H, Ar), 7.41–7.39 (m, 1H, Ar), 7.39–7.37 (m, 2H, Ar), 7.37–7.34 (m, 3H, Ar), 7.34–7.31 (m, 1H, Ar), 7.21 (t, *J* = 7.7 Hz, 2H, Ar), 7.13 (d, *J* = 8.2 Hz, 2H, Ar), 5.48 (s, 2H, CH_2_–C=O), 5.23 (s, 2H, CH_2_–O);^13^C NMR (126 MHz, DMSO-*d*_6_) *δ* 164.82 (NH–C=O), 159.12 (–N=C–N=), 158.61, 156.31, 150.59, 143.91, 142.28, 136.15, 135.98 (N=CH), 134.13, 130.15, 129.57, 129.36, 128.97, 128.30, 128.19, 127.51, 126.66, 126.35, 126.24, 125.82, 115.01, 61.12 (CH_2_–O), 51.94 (CH_2_–C=O); anal. calcd: C_33_H_26_ClN_9_O_2_; C, 64.34; H, 4.25; N, 20.46; found: C, 64.39; H, 4.36; N, 20.52.

### (*E*)-*N*-(3-Chlorophenyl)-2-(4-((4-((2-(5,6-diphenyl-1,2,4-triazin-3-yl)hydrazineylidene)methyl)phenoxy)methyl)-1*H*-1,2,3-triazol-1-yl)acetamide (13i)

Yellowish white solid; yield: 86%; MP = 280–282 °C; ^1^H NMR (500 MHz, DMSO-*d*_6_) *δ* 11.82 (s, 1H, NH, NH–N=), 10.07 (s, 1H, NH–C=O), 8.28 (s, 1H, N=CH), 8.24 (s, 1H, triazole), 7.74 (d, *J* = 6.5 Hz, 1H, Ar), 7.68 (d, *J* = 8.2 Hz, 2H, Ar), 7.52 (d, *J* = 6.6 Hz, 1H, Ar), 7.48 (d, *J* = 7.1 Hz, 2H, Ar), 7.44 (d, *J* = 7.3 Hz, 1H, Ar), 7.41–7.40 (m, 1H, Ar), 7.38 (d, *J* = 8.2 Hz, 2H, Ar), 7.36–7.35 (m, 3H, Ar), 7.33 (d, *J* = 7.4 Hz, 1H, Ar), 7.21 (t, *J* = 7.0 Hz, 1H, Ar), 7.14 (d, *J* = 8.3 Hz, 2H, Ar), 5.47 (s, 2H, CH_2_–C=O), 5.23 (s, 2H, CH_2_–O);^13^C NMR (126 MHz, DMSO-*d*_6_) *δ* 164.82 (NH–C=O), 159.13 (–N=C–N=), 158.57, 156.31, 150.58, 143.89, 142.27, 136.15, 135.98 (N=CH), 134.12, 130.16, 129.58, 129.37, 128.98, 128.32, 128.21, 128.14, 127.64, 127.52, 126.68, 126.35, 126.26, 125.84, 115.04, 61.11 (CH_2_–O), 51.92 (CH_2_–C=O); anal. calcd: C_33_H_26_ClN_9_O_2_; C, 64.34; H, 4.25; N, 20.46; found: C, 64.49; H, 4.32; N, 20.58.

### (*E*)-*N*-(4-Chlorophenyl)-2-(4-((4-((2-(5,6-diphenyl-1,2,4-triazin-3-yl)hydrazineylidene)methyl)phenoxy)methyl)-1*H*-1,2,3-triazol-1-yl)acetamide (13j)

Brown solid; yield: 85%; MP = 295–297 °C; ^1^H NMR (500 MHz, DMSO-*d*_6_) *δ* 11.84 (s, 1H, NH, NH–N=), 10.08 (s, 1H, NH–C=O), 8.28 (s, 1H, N=CH), 8.24 (s, 1H, triazole), 7.74 (d, *J* = 8.0 Hz, 1H, Ar), 7.68 (d, *J* = 8.2 Hz, 2H, Ar), 7.52 (d, *J* = 7.9 Hz, 1H, Ar), 7.49–7.46 (m, 2H, Ar), 7.44 (d, *J* = 7.2 Hz, 1H, Ar), 7.42–7.40 (m, 1H, Ar), 7.39–7.38 (m, 2H, Ar), 7.38–7.36 (m, 2H, Ar), 7.36–7.35 (m, 2H, Ar), 7.35–7.34 (m, 1H, Ar), 7.33 (d, *J* = 7.8 Hz, 1H, Ar), 7.21 (t, *J* = 7.7 Hz, 1H, Ar), 7.13 (d, *J* = 8.1 Hz, 1H, Ar), 5.47 (s, 2H, CH_2_–C=O), 5.23 (s, 2H, CH_2_–O); ^13^C NMR (126 MHz, DMSO-*d*_6_) *δ* 164.82 (NH–C=O), 159.13 (–N=C–N=), 158.58, 156.32, 150.59, 143.89, 142.26, 136.14, 135.98 (N=CH), 134.12, 130.16, 129.58, 129.36, 128.98, 128.33, 128.21, 127.52, 126.68, 126.35, 125.84, 61.11 (CH_2_–O), 51.92 (CH_2_–C=O); anal. calcd: C_33_H_26_ClN_9_O_2_; C, 64.34; H, 4.25; N, 20.46; found: C, 64.41; H, 4.39; N, 20.51.

### (*E*)-*N*-(4-Bromophenyl)-2-(4-((4-((2-(5,6-diphenyl-1,2,4-triazin-3-yl)hydrazineylidene)methyl)phenoxy)methyl)-1*H*-1,2,3-triazol-1-yl)acetamide (13k)

Yellow solid; yield: 87%; MP = 279–280 °C; ^1^H NMR (500 MHz, DMSO-*d*_6_) *δ* 11.85 (s, 1H, NH, NH–N=), 10.65 (s, 1H, NH–C=O), 8.29 (s, 1H, N=CH), 8.26 (s, 1H, triazole), 7.69 (d, *J* = 8.2 Hz, 2H, Ar), 7.56 (d, *J* = 8.5 Hz, 2H, Ar), 7.51 (d, *J* = 8.6 Hz, 2H, Ar), 7.48 (d, *J* = 7.3 Hz, 2H, Ar), 7.43 (d, *J* = 7.3 Hz, 1H, Ar), 7.38 (d, *J* = 7.9 Hz, 2H, Ar), 7.37–7.33 (m, 4H, Ar), 7.13 (d, *J* = 8.2 Hz, 2H, Ar), 5.38 (s, 2H, CH_2_–C=O), 5.23 (s, 2H, CH_2_–O); ^13^C NMR (126 MHz, DMSO-*d*_6_) *δ* 164.34 (NH–C=O), 159.12 (–N=C–N=), 158.53, 156.31, 150.56, 143.93, 142.27, 137.73, 136.15, 135.98 (N=CH), 131.70, 130.15, 129.37, 128.97, 128.30, 128.19, 127.64, 126.32, 121.16, 115.40, 115.01, 61.12 (CH_2_–O), 52.22 (CH_2_–C=O); anal. calcd: C_33_H_26_BrN_9_O_2_; C, 60.01; H, 3.97; N, 19.08; found: C, 60.16; H, 4.11; N, 19.17.

### (*E*)-2-(4-((4-((2-(5,6-Diphenyl-1,2,4-triazin-3-yl)hydrazineylidene)methyl)phenoxy)methyl)-1*H*-1,2,3-triazol-1-yl)-*N*-(2-nitrophenyl)acetamide (13l)

Yellow solid; yield: 85%; MP = 278–279 °C; ^1^H NMR (500 MHz, DMSO-*d*_6_) *δ* 11.86 (s, 1H, NH, NH–N=), 10.78 (s, 1H, NH–C=O), 8.28–8.24 (m, 2H, N=CH and triazole), 7.98 (d, *J* = 7.9 Hz, 2H, Ar), 7.73 (d, *J* = 8.1 Hz, 2H, Ar), 7.71–7.68 (m, 2H, Ar), 7.49–7.46 (m, 2H, Ar), 7.45–7.42 (m, 2H, Ar), 7.42–7.40 (m, 2H, Ar), 7.39–7.38 (m, 2H, Ar), 7.38–7.34 (m, 2H, Ar), 7.18–7.11 (m, 2H, Ar), 5.46 (s, 2H, CH_2_–C=O), 5.23 (s, 2H, CH_2_–O); ^13^C NMR (126 MHz, DMSO-*d*_6_) *δ* 164.90 (NH–C=O), 159.15 (–N=C–N=), 156.37, 156.30, 150.55, 143.98, 142.32, 136.12, 136.06 (N=CH), 134.13, 130.34, 130.17, 129.36, 128.99, 128.34, 128.21, 126.34, 125.84, 125.49, 125.03, 115.02, 61.11 (CH_2_–O), 51.97 (CH_2_–C=O); anal. calcd: C_33_H_26_N_10_O_4_; C, 63.25; H, 4.18; N, 22.35; found: C, 63.37; H, 4.22; N, 22.40.

### (*E*)-*N*-Benzyl-2-(4-((4-((2-(5,6-diphenyl-1,2,4-triazin-3-yl)hydrazineylidene)methyl)phenoxy)methyl)-1*H*-1,2,3-triazol-1-yl)acetamide (13m)

Milky white solid; yield: 86%; MP = 272–273 °C; ^1^H NMR (500 MHz, DMSO-*d*_6_) *δ* 11.86 (s, 1H, NH, NH–N=), 8.86 (t, *J* = 5.9 Hz, 1H, NH–C=O), 8.26 (s, 1H, N=CH), 8.24 (s, 1H, triazole), 7.69 (d, *J* = 8.2 Hz, 2H, Ar), 7.48 (d, *J* = 7.4 Hz, 2H, Ar), 7.43 (d, *J* = 7.3 Hz, 1H, Ar), 7.42–7.38 (m, 2H, Ar), 7.40–7.36 (m, 1H, Ar), 7.38–7.34 (m, 1H, Ar), 7.36–7.33 (m, 3H, Ar), 7.32 (d, *J* = 7.2 Hz, 2H, Ar), 7.31–7.26 (m, 2H, Ar), 7.26 (d, *J* = 7.0 Hz, 1H, Ar), 7.13 (d, *J* = 8.1 Hz, 2H, Ar), 5.22 (s, 2H, CH_2_–C=O), 5.21 (s, 2H, CH_2_–O), 4.34 (d, *J* = 6.0 Hz, 2H, C**H_2_**–NH); ^13^C NMR (126 MHz, DMSO-*d*_6_) *δ* 170.26 (NH–C=O), 165.37 (–N=C–N=), 159.15, 158.59, 156.32, 150.56, 143.94, 142.17, 138.65, 136.15, 135.98 (N=CH), 130.16, 129.38, 128.98, 128.31, 128.20, 127.63, 127.36, 126.96, 126.20, 115.01, 61.13 (CH_2_–O), 51.62 (CH_2_–C=O), 42.38 (CH_2_–NH); anal. calcd: C_34_H_29_N_9_O_2_; C, 68.56; H, 4.91; N, 21.16; found: C, 68.72; H, 4.98; N, 21.29.

### (*E*)-2-(4-((4-((2-(5,6-Diphenyl-1,2,4-triazin-3-yl)hydrazineylidene)methyl)phenoxy)methyl)-1*H*-1,2,3-triazol-1-yl)-*N*-phenethylacetamide (13n)

Yellow solid; yield: 85%; MP = 273–274 °C; ^1^H NMR (500 MHz, DMSO-*d*_6_) *δ* 11.83 (s, 1H), 8.44 (t, *J* = 5.6 Hz, 1H), 8.24 (s, 1H), 8.17 (s, 1H), 7.68 (d, *J* = 8.2 Hz, 2H, Ar), 7.48 (d, *J* = 7.0 Hz, 2H, Ar), 7.44 (d, *J* = 7.3 Hz, 1H, Ar), 7.41–7.40 (m, 1H, Ar), 7.38 (d, *J* = 8.0 Hz, 2H, Ar), 7.37–7.34 (m, 3H, Ar), 7.30 (d, *J* = 7.0 Hz, 1H, Ar), 7.28 (d, *J* = 7.0 Hz, 1H, Ar), 7.22–7.19 (m, 3H, Ar), 7.13 (d, *J* = 8.2 Hz, 2H, Ar), 5.21 (s, 2H, CH_2_–C=O), 5.09 (s, 2H, CH_2_–O), 3.32 (m, 2H, **CH_2_**–NH), 2.74 (t, *J* = 7.4 Hz, 2H, **CH_2_**-Ph); ^13^C NMR (126 MHz, DMSO-*d*_6_) *δ* 165.19 (NH–C=O), 156.30 (–N=C–N=), 150.58, 147.60, 143.87, 142.14, 139.12, 136.16, 135.98 (N=CH), 130.16, 129.37, 128.98, 128.58, 128.31, 128.21, 128.12, 127.64, 126.12, 126.09, 115.03, 61.11 (CH_2_–O), 51.62 (CH_2_–C=O), 40.35 (**C**H_2_–NH), 34.86 (**C**H_2_-Ph); anal. calcd: C_35_H_31_N_9_O_2_; C, 68.95; H, 5.13; N, 20.68; found: C, 69.04; H, 5.28; N, 20.71.

### Anti-α-glucosidase assay

α-Glucosidase (EC3.2.1.20, *Saccharomyces cerevisiae*, 20 U mg^−1^) and *p*-nitrophenyl-α-d-glucopyranoside (substrate) were purchased from Sigma-Aldrich. α-Glucosidase was prepared in potassium phosphate buffer (KPB, pH 6.8, 50 mM), and the target compounds 13a–n were dissolved in dimethyl sulfoxide (DMSO, 10% final concentration, negative control). The 135 μL of KPB, 20 μL of various concentrations of compounds 13a–n, and 20 μL of α-glucosidase solution were added in the 96-well plate and incubated for 10 min at 37 °C. After this time, the *p*-nitrophenyl-α-d-glucopyranoside (25 μL, 4 mM) was added to the latter plate and allowed to incubate at 37 °C for 20 min. After that, the change in absorbance was measured at 405 nm by using a spectrophotometer (Gen5, Power wave xs2, BioTek, America). Acarbose was used as the positive control. The percentage of inhibition (% inhibition) for each sample was calculated using the formula “[(Abs control − Abs sample)/Abs control] × 100”, and IC_50_ values were obtained from non-linear regression curve using the Logit method.^24^

### Kinetic study

Kinetic analysis was performed on the best compound (determined by *in vitro* study) using the Lineweaver–Burk method. The selected compound at various concentrations (0, 30, 60, 120, and 240 nM) was added to the enzyme solution (1 U mL^−1^) at 37 °C and incubated for 10 min. Then, *p*-nitrophenyl-α-d-glucopyranoside at various concentrations (1–4 mM) was added to initiate the enzymatic reaction. Changes in absorbance were measured at 405 nm for 20 minutes using a spectrophotometer.^24^

### Statistical analysis

All data were organized and analyzed using Microsoft Excel. Descriptive statistics were applied to calculate mean values and standard deviations, which are reported in the Table 1.

**Table 1:** Anti-α-glucosidase activity of target compounds 13a–n

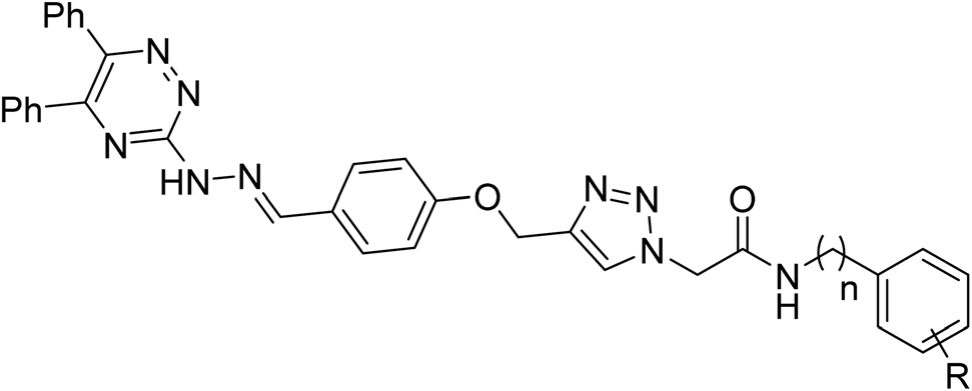			
Compound	*n*	R	IC_50_^a^ (μM)
13a	0	H	0.8 ± 0.04
13b	0	2-Me	12.5 ± 0.21
13c	0	3-Me	1.8 ± 0.02
13d	0	2,4-Dimethyl	2.5 ± 0.12
13e	0	4-Et	3.7 ± 0.13
13f	0	4-OMe	10.9 ± 0.24
13g	0	2-F	0.3 ± 0.11
13h	0	2-Cl	0.19 ± 0.05
13i	0	3-Cl	11.7 ± 0.18
13j	0	4-Cl	0.12 ± 0.02
13k	0	4-Br	15.3 ± 0.29
13l	0	2-NO_2_	34.2 ± 0.57
13m	1	H	0.9 ± 0.07
13n	2	H	0.5 ± 0.06
Acarbose	—	—	750.0 ± 0.85

a Values are the mean ± SD. All experiments were performed at least three times.

### Docking study

Molecular docking of the most potent compounds was performed using a homology-modeled structure of *S. cerevisia* α-glucosidase, since no crystal structure was available in the Protein Data Bank (PDB).^24^ This model was generated with SWISS-MODEL based on *S. cerevisiae* isomaltase (PDB: 3A4A, 72% identity, 85% similarity) and validated by PROCHECK. The 3D structures of the selected compounds were built in MarvinSketch, converted to pdbqt format in AutoDock Tools, and docked into the active site using AutoDock with a grid box of 40 × 40 × 40 Å and 0.375 Å spacing. Fifty runs of the Lamarckian genetic algorithm were carried out for each selected compound, and the best binding poses were analyzed with BIOVIA Discovery Studio (Fig. 7).

### Molecular dynamics

Molecular dynamics (MD) simulations of α-glucosidase in complex with acarbose and the most active ligand were performed using the GROMACS 5.1.2 package on an Ubuntu 18.04.5 LTS platform.^31^ The topologies and force-field parameters for the ligands were prepared using the SwissParam web server, which provides CHARMM-compatible parameters for small organic molecules suitable for both CHARMM and GROMACS.^32^ The protein topology was generated with the pdb2gmx utility in GROMACS, applying the CHARMM36 all-atom force field. Ligand and protein coordinate files were converted into GROMACS format (.gro) and combined manually in Notepad++. The protein topology file (.top) was modified to include the ligand-specific parameters by referencing the corresponding.itp file from SwissParam. The resulting protein–ligand system was placed at the center of a cubic box, ensuring at least 1.0 nm between the complex and the box boundaries. The system was solvated using the SPC216 water model, and overall charge neutrality was achieved by replacing fifteen water molecules with sodium ions (Na^+^), compensating for the protein's net negative charge.

Energy minimization was carried out with the steepest descent algorithm for up to 50 000 steps or until the maximum force fell below 10.0 kJ mol^−1^. The minimized system was equilibrated in two stages: (i) under an NVT ensemble at 300 K for 500 ps using the velocity-rescaling thermostat with a coupling constant of 0.1 ps, followed by (ii) NPT equilibration at 1 bar for 1000 ps using the Berendsen barostat with a pressure coupling constant of 5.0 ps. Long-range electrostatics were treated with the Particle Mesh Ewald (PME) method, while short-range electrostatic and van der Waals interactions were handled using cut-off distances of 1.0 nm and 1.2 nm, respectively. Finally, a 20 ns production MD simulation was performed to investigate the conformational stability and dynamic behavior of the protein–ligand complexes.

### *In silico* study on pharmacokinetics

For calculations in this section, we used of SwissADME, pkCSM, PreADMET, and admetSAR, as four reliable online software tools.^33–36^ For SwissADME, pkCSM, and admetSAR, the selected compounds were copied as the SMILES format in ChemDraw 18.2 and were inserted in https://www.swissadme.ch/, https://biosig.lab.uq.edu.au/pkcsm/, and https://lmmd.ecust.edu.cn/admetsar3/predict.php, respectively, as input files. For PreADMET, the Mol files of the selected compounds were prepared using ChemDraw 18.2 and uploaded in https://preadmet.webservice.bmdrc.org as input file.

## Result and discussion

### Chemistry

**Fig. 2:**
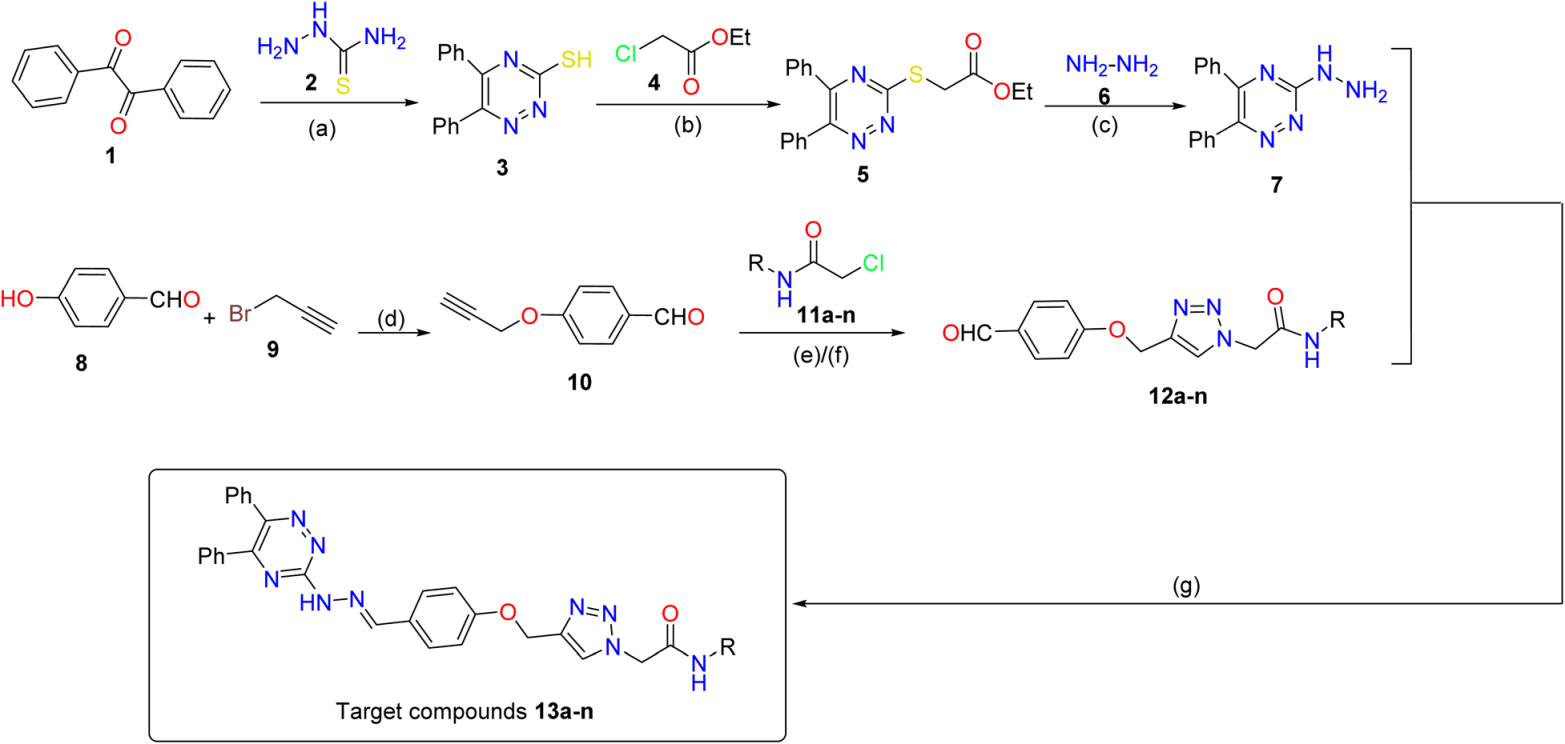
Synthesis of 1,2,4-triazine-hydrazineylidene-phenoxy-1,2,3-triazole-acetamide derivatives 13a–n: (a) acetic acid, Reflux, 6 h; (b) DMF, K_2_CO_3_, RT, 12 h; (c) ethanol, 60 °C, 24 h; (d) DMF, K_2_CO_3_, RT, 8 h; (e) NaN_3_, NEt_3_, H_2_O/*t*-BuOH, 1 h; (f) CuSO_4_·5H_2_O, sodium ascorbate, RT, 24–48 h; (g) ethanol, acetic acid, 60 °C, 12 h.

For the synthesis of the new 5,6-diphenyl-1,2,4-triazine-hydrazineylidene-phenoxy-1,2,3-triazole-acetamide derivatives 13a–n, we employed the method depicted in Fig. 2. To produce the title compounds, 3-hydrazineyl-5,6-diphenyl-1,2,4-triazine (7) and 2-(4-((4-formylphenoxy)methyl)-1*H*-1,2,3-triazol-1-yl)acetamide derivatives 12a–n were used as starting material. Compound 7 was obtained by following reactions: reaction between benzil (1) and hydrazinecarbothioamide (2) to give 5,6-diphenyl-1,2,4-triazine-3-thiol (3), reaction between the latter compound 3 with ethyl 2-chloroacetate (4) to give ethyl 2-((5,6-diphenyl-1,2,4-triazine-3-yl)thio)acetate (5), and reaction between compound 5 with hydrazine (6) to give compound 7. On the other hand, compounds 12a–n were synthesized using the reported methods described in our previous works.^21^ In the last step, compound 7 was reacted with compounds 12a–n to give title compounds 13a–n.

**Fig. 3:**
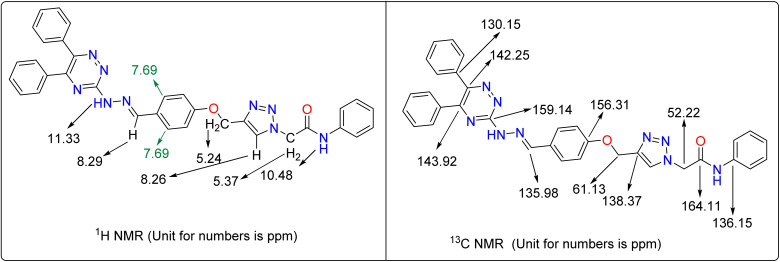
^1^H NMR and ^13^C NMR interpretations of compound 13a.

^1^H NMR and ^13^C NMR interpretations of compound 13a, for instance, are schematically shown in Fig. 3.

### *In vitro* α-glucosidase inhibition assay

The target compounds 13a–n were evaluated against the yeast form of α-glucosidase, which was extracted from *Saccharomyces cerevisiae*. All the compounds 13a–n exhibited significant inhibitory activity against the studied form of α-glucosidase compared with the standard inhibitor, acarbose. In this regard, the most potent compound was 13j, exhibiting inhibitory activity 6250 times greater than acarbose, while the least potent compound was 13l, with inhibitory activity 21.9 times greater than acarbose.

### Structure–activity relationships (SARs)

**Fig. 4:**
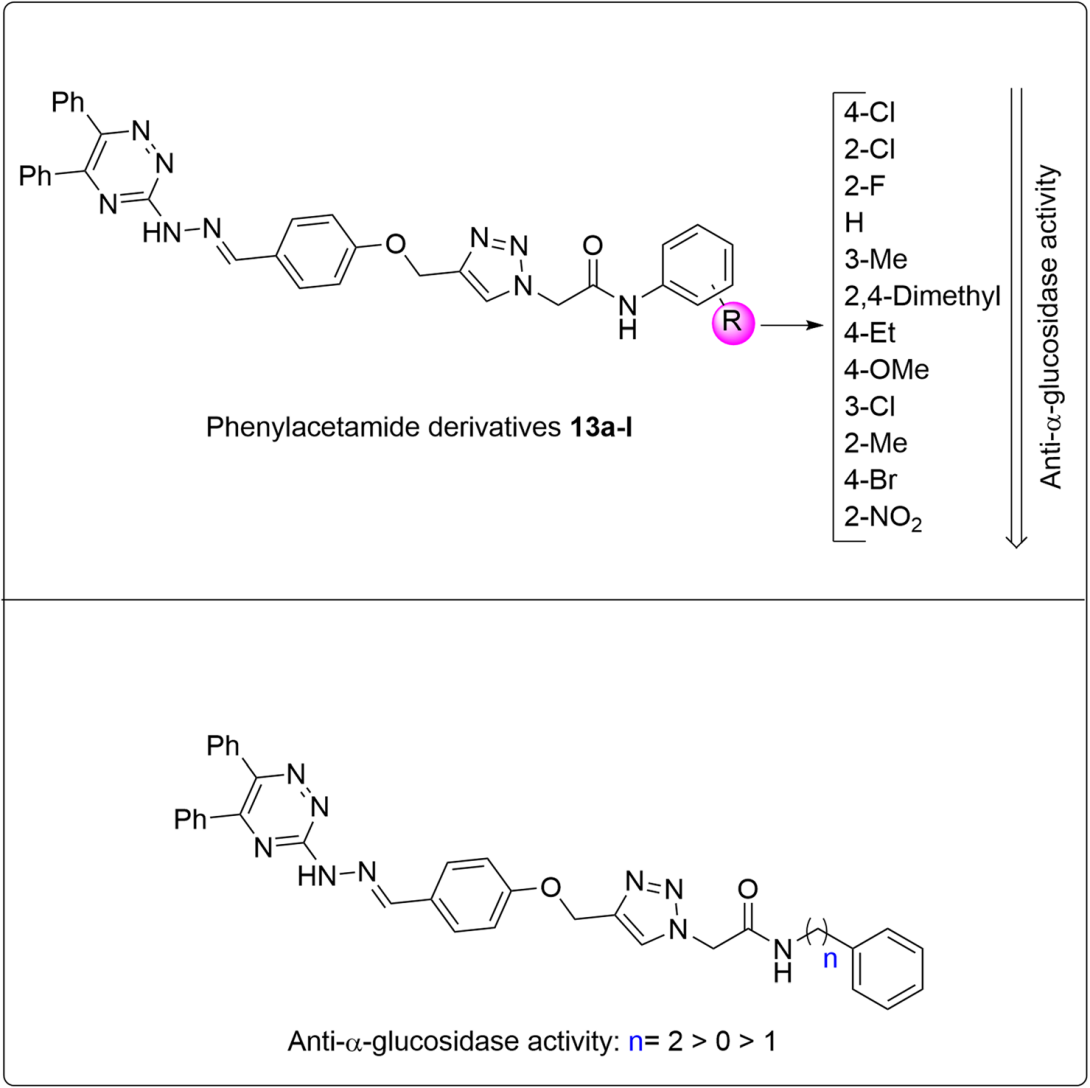
SAR diagram of new compounds 13a–n in the enzymatic inhibition assay.

As can be seen in Fig. 2, derivatization in the present work is based on modifications in the acetamide moiety. In this regard, we have twelve *N*-phenylacetamide derivatives (compounds 13a–l), a *N*-benzylacetamide derivative (compound 13m), and a *N*-phenethylacetamide derivative (compound 13n). SAR diagrams of these compounds were showed in Fig. 4.

As can be seen in the SAR diagram of *N*-phenylacetamide derivatives 13a–l (Fig. 4a), the first, second, and third potent compounds were 4-chloro (compound 13j), 2-chloro (compound 13h), and 2-fluoro (compound 13g), respectively. These compounds were also the most potent compounds among all the synthesized compounds 13a–n. Moreover, unsubstituted compound 13a in this series was a significant inhibitor against α-glucosidase. This compound was the fifth potent compound among the compounds 13a–n. The addition of a methyl group at the 2-position of the phenyl ring in the un-substituted derivative 13a decreased its inhibitory activity to 15.6-fold, while the same substituent at the 3-position reduced anti-α-glucosidase activity to 2.25-fold, as observed in compounds 13b and 13c, respectively. This finding indicated that the position of substituents plays an important role in anti-α-glucosidase activity. On the other hand, 4-ethyl derivative 13e was around 3-fold more potent than 4-methoxy derivative 13f. Therefore, even among derivatives with electron-donating substituents, the type of substitution is very decisive in the obtained inhibition effect. As shown in Table 1, this trend is also observed in derivatives with electron-withdrawing substituents (4-chloro derivative 13j*vs.* 4-bromo derivative 13k, and 2-fluoro derivative 13g and 2-chloro derivative 13h*vs.* 2-nitro derivative 13l). In addition to the mentioned points, comparison of the IC_50_ values of the 2-methyl derivative 13b with its 2,4-dimethyl analog 13d indicates that the introduction of a second methyl group significantly increased α-glucosidase inhibition potency.

Table 1 and Fig. 4b also show that the *N*-phenylacetamide derivative 13a, the *N*-benzylacetamide derivative 13m, and the *N*-phenethylacetamide derivative 13n are highly potent against α-glucosidase, with the most active being phenethyl derivative (compound 13n).

### Comparison of the anti-α-glucosidase activities of the new compounds 13 with used templates

**Fig. 5:**
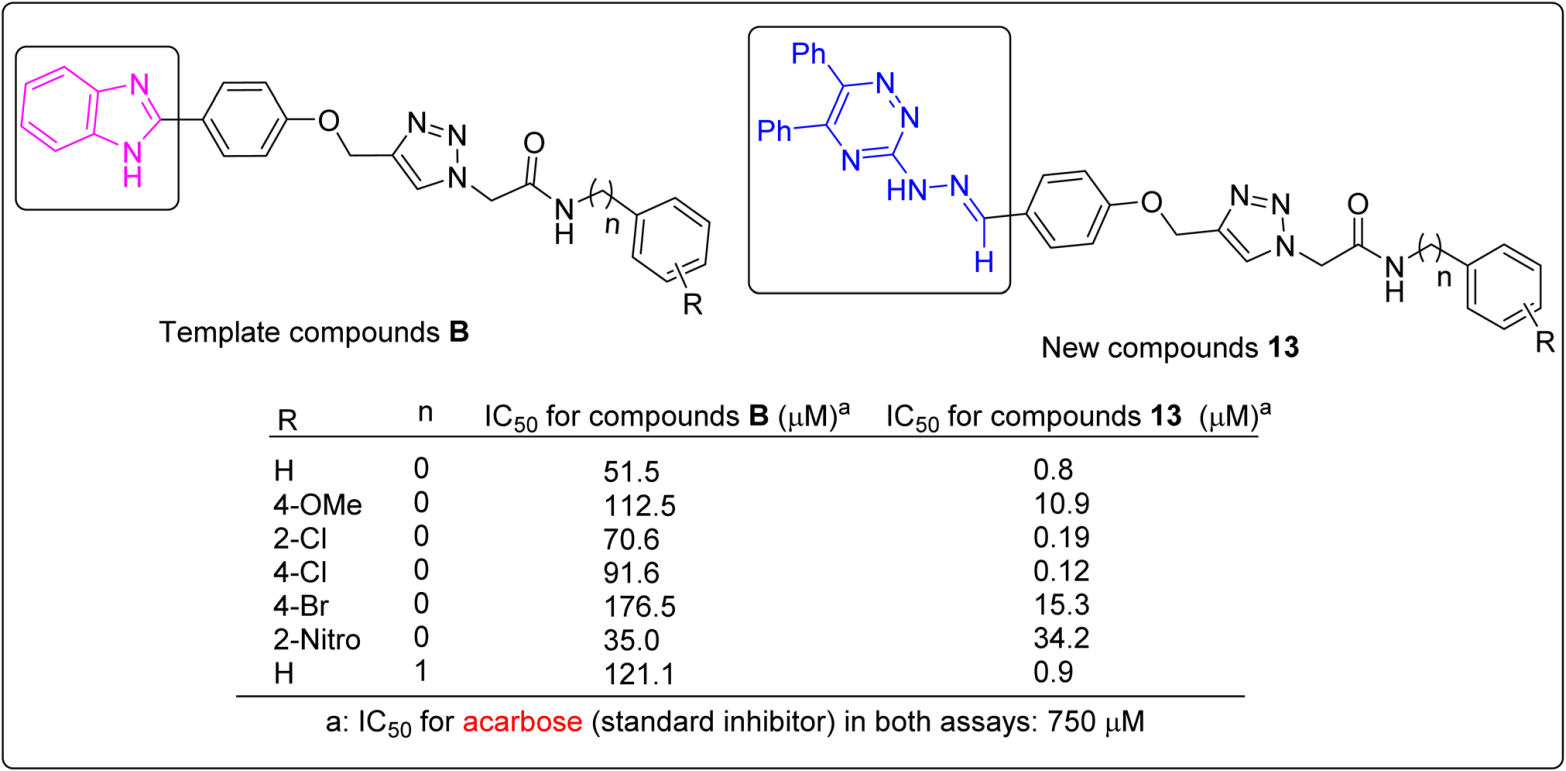
Comparison of the template compounds B and their corresponding analogs of compounds 13 in anti-α-glucosidase assay.

In this section, α-glucosidase inhibitory activities of the new 5,6-diphenyl-1,2,4-triazine-hydrazineylidene-phenoxy-1,2,3-triazole-acetamide derivatives 13 were compared with the templates that used for designing these compounds. A simple review of Fig. 1 shows that our new compounds are significantly more potent than 5,6-diphenyl-1,2,4-triazine-hydrazineylidene derivatives A.^21^ But comparing compounds 13 with phenoxy-1,2,3-triazole-acetamide derivatives B is a bit more complicated.^24^ For this reason, we performed a more detailed comparison and reported compounds B were compared to their corresponding analogs of the new compounds 13. The obtained results of this comparison is shown in Fig. 5. This figure showed that our new compounds were significantly more potent than their corresponding analogs of compounds B.

### Kinetic study

**Fig. 6:**
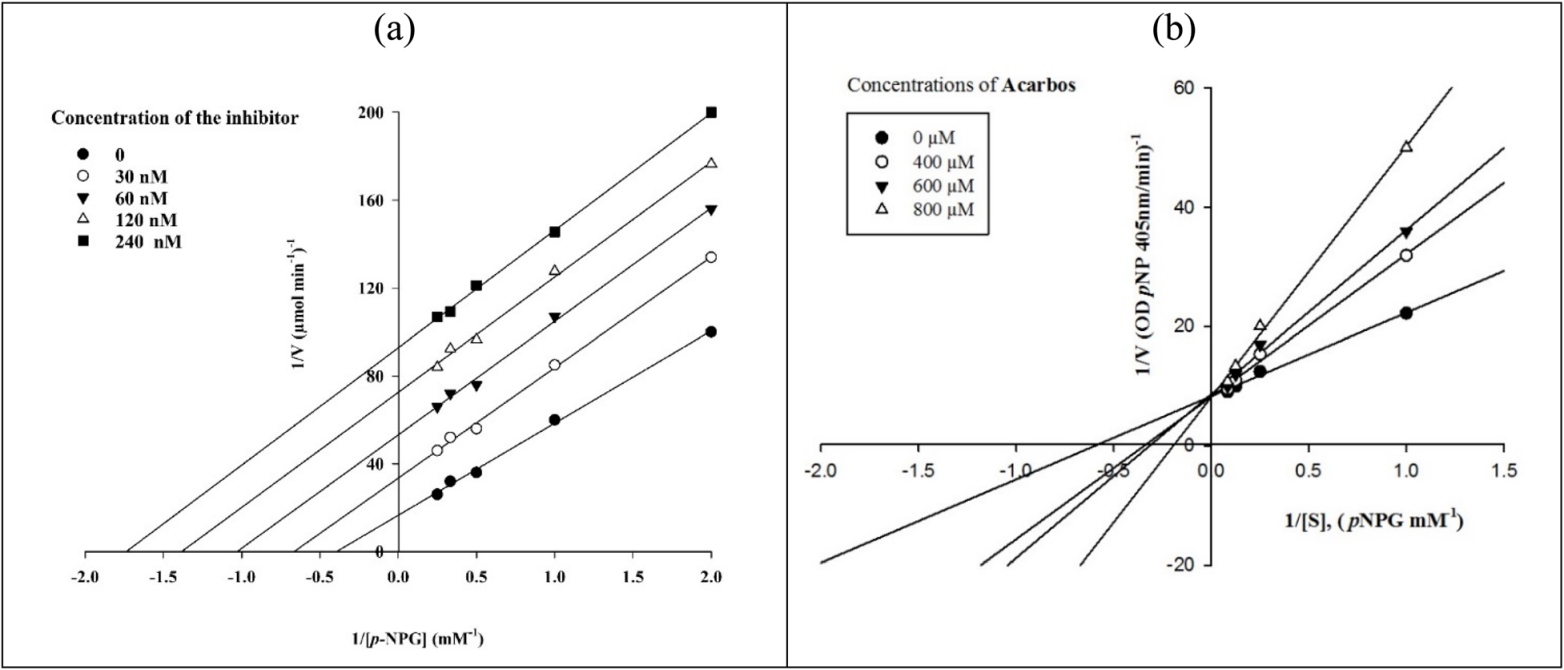
The Lineweaver–Burk plots in the absence and presence of the different concentrations of new compound 13j (a) and acarbose (b).

To investigate the enzymatic inhibition mode of the newly synthesized compounds against α-glucosidase, kinetic studies were performed on the most potent compound 13j and the standard inhibitor. The type of inhibition mode was indicated by Lineweaver–Burk plots. As shown in Fig. 6a and b, increasing the concentration of compound 13j resulted in decrease in both *V*_max_ and *K*_m_ values, whereas increasing the concentration of acarbose did not change *V*_max_ but increased *K*_m_. These findings indicated that compound 13j is an uncompetitive inhibitor and acarbose is a competitive inhibitor.

### Docking study

**Fig. 7:**
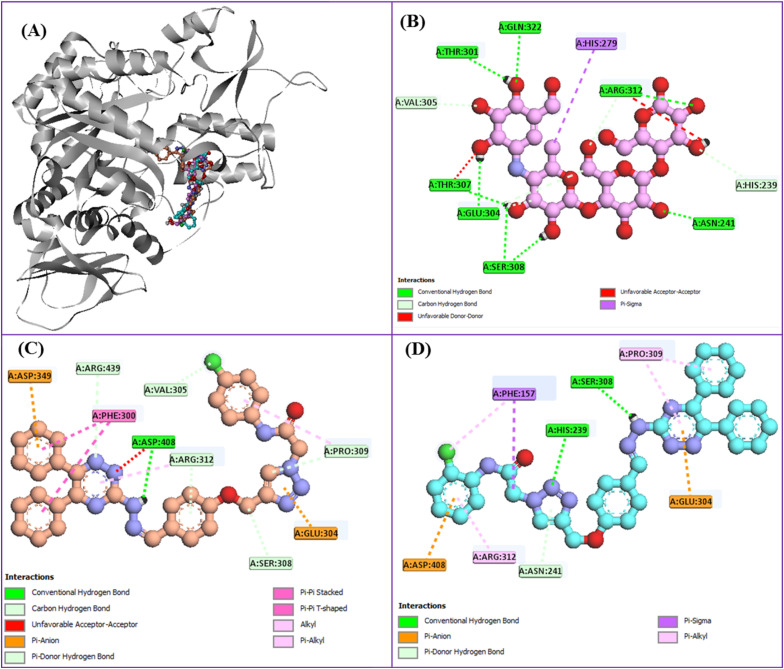
Superimposed conformation of acarbose (pink), compound 13j (orange), and compound 13h (cyan) within the α-glucosidase active site (A). 2D interaction mode of acarbose (B), compound 13j (C), and compound 13h (D) in the latter site.

In addition to the SAR analysis based on *in vitro* data, an *in silico* docking study was conducted on the most active compounds, 13j and 13h.^24^ In the first step of our docking study, the reference inhibitor acarbose and compounds 13j and 13h were docked in the active site of α-glucosidase and their superimposed structure was showed in Fig. 7A.

Docking analysis of acarbose revealed the following interactions within the α-glucosidase active site: (i) hydrogen bonds with Thr301, Gln322, Thr307, Arg312, Glu304, Ser308, and Asn241; (ii) a non-classical hydrogen bond with His239; (iii) a hydrophobic contact with His279; and (iv) unfavorable interactions with Arg312 and Thr207 (Fig. 7B).

As illustrated in Fig. 7C, the most active derivative, compound 13j, established a classical hydrogen bond along with an unfavorable interaction with Asp408. Additionally, this compound formed two π-anion interactions with Asp439 and Glu304, as well as two π–π stacking interactions with Phe300. Several non-classical hydrogen bonds were also observed between compound 13j and residues Arg439, Val305, Pro309, Arg312, and Ser308. Moreover, two hydrophobic interactions were detected with Pro309 and Arg312.

The interaction profile of the second most potent compound 13h is depicted in Fig. 7D. This compound formed two classical hydrogen bonds with residues Ser308 and His239, along with a non-classical hydrogen bond with Asn241. In addition, compound 13h established two π-anion interactions with Glu304 and Asp408 and four hydrophobic interactions with residues Pro309 (two interactions), Phe157, and Arg312.

The calculated binding energies for standard inhibitor acarbose (−4.4 kcal mol^−1^), compound 13j (−11.29 kcal mol^−1^), and compound 13h (−9.1 kcal mol^−1^) indicated that both newly synthesized compounds exhibited substantially stronger binding affinities compared to acarbose. Moreover, the most potent compound 13j demonstrated the more favorable binding energy than the second potent compound 13h, which was consistent with the results obtained from the *in vitro* enzymatic inhibition assays.

### Molecular dynamics

The binding of a ligand to a receptor is highly dynamic, typically occurring on sub-nanosecond timescales. To better understand the conformational stability and dynamic properties of receptor–ligand complexes, molecular simulations under conditions that closely resemble the physiological environment are particularly valuable, usually incorporating explicit water molecules and appropriate ions. In this work, molecular dynamics (MD) simulations were performed in a fully solvated system to explore the dynamic behavior of acarbose, taken as a reference inhibitor, along with compound 13j, which was identified as the most effective α-glucosidase inhibitor *in vitro*. The goal of these simulations was to evaluate the structural stability and flexibility of protein-inhibitor complexes throughout the simulation trajectory.

**Fig. 8:**
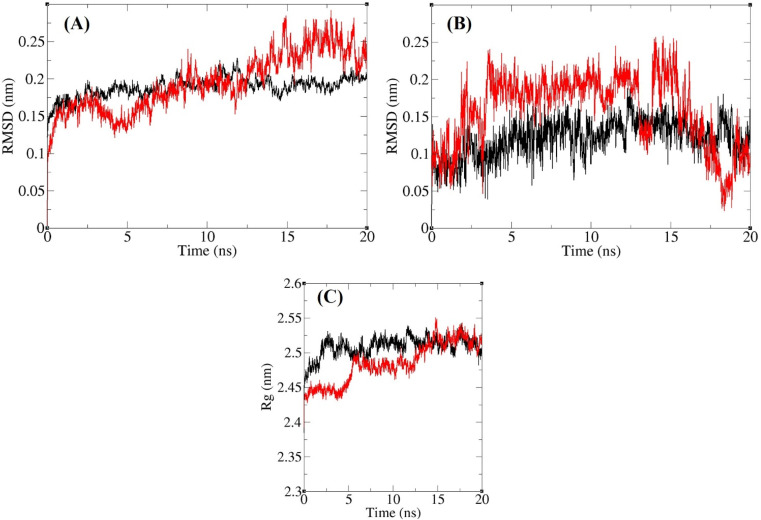
Superimposed RMSD of Cα atoms of α-glucosidase in complex with compound 13j (red) and acarbose (black) (A). Superimposed RMSD of compound 13j (red) and acarbose (black) in complex with α-glucosidase (B). Time dependence of the radius of gyration (*R*_g_) graph of α-glucosidase in complex with compound 13j (red) and acarbose (black) (C).

**Fig. 9:**
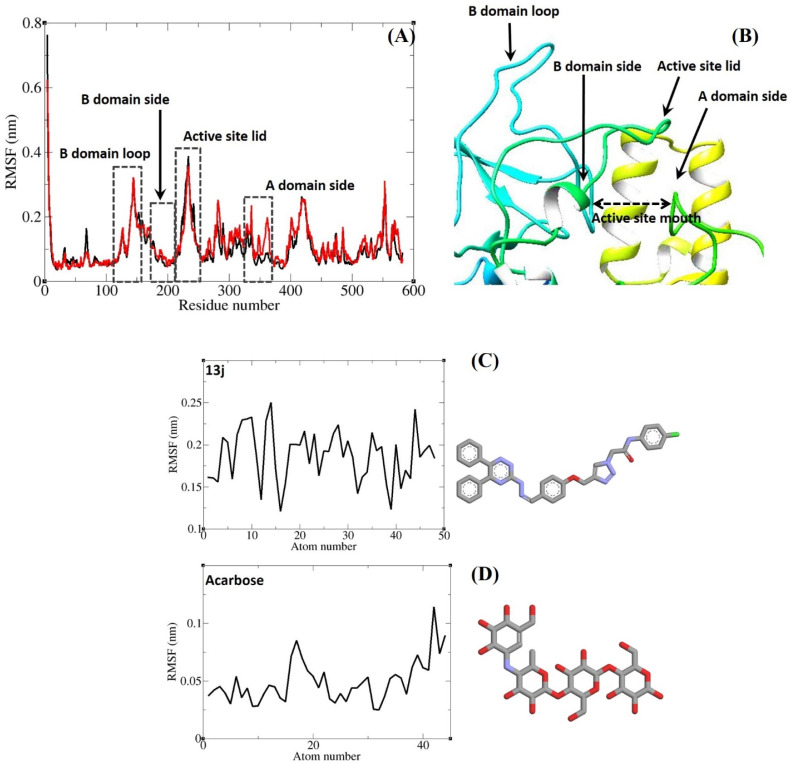
RMSF graph of Cα atoms of α-glucosidase in complex with compound 13j (red) and acarbose (black) (A). Close-up representation of α-glucosidase active site (B). RMSF graph of the heavy atoms of compound 13j (C) and acarbose (D) in complex with α-glucosidase. Structure of these compounds are illustrated.

Two runds of MD simulations were carried out. Initially, each protein–ligand complex underwent a 10 ns simulation, during which both acarbose and compound 13j maintained stable interactions within the α-glucosidase active site. To obtain a more detailed picture of their dynamic behavior, the simulations were extended for an additional 10 ns. The extended trajectories confirmed that both ligands continued to exhibit stable binding throughout the entire run. The generated trajectories were subsequently analyzed with several computational approaches. Root-mean-square deviation (RMSD) and radius of gyration (*R*_g_) values were calculated for all sampled conformations to track structural fluctuations and assess overall stability of the complexes over time. In addition, the root-mean-square fluctuation (RMSF) of backbone atoms was examined to evaluate residue-specific flexibility during the simulation. The outcomes of these analyses are summarized in Fig. 8 and 9. As shown in Fig. 8, the RMSD values of α-glucosidase remained steady, staying below 0.30 nm across the entire simulation. The mean RMSD values of α-glucosidase in complex with either acarbose or compound 13j were 0.19 nm and 0.18 nm, respectively. Indicating negligible structural deviations and confirming complex stability. Similarly, RMSD values for acarbose and compound 13j within the binding pocket were consistently below 0.25 nm, with no notable fluctuations during the simulation (Fig. 8). Their corresponding mean RMSD values were 0.12 nm for acarbose and 0.16 nm for compound 13j. Taken together, these findings demonstrate that both ligands retained stable binding conformations within the α-glucosidase active site throughout the MD simulations.

The structural compactness of α-glucosidase during the simulations was evaluated through the calculation of its radius of gyration (*R*_g_), as depicted in Fig. 8. Across the trajectory, the Rg values of the enzyme bound to either acarbose or compound 13j consistently fell within a narrow window of 2.39–2.55 nm. The absence of large variations or long-term shifts indicates that the tertiary fold of the protein remained intact. The average *R*_g_ values obtained were 2.50 nm for the acarbose complex and 2.48 nm for the compound 13j complex, confirming that ligand association did not trigger noticeable structural expansion or contraction.

RMSF analysis of Cα atoms was performed to probe residue-level flexibility (Fig. 9). The RMSF profiles of α-glucosidase complexed with either acarbose or compound 13j displayed very similar patterns. Given that α-glucosidase is a large multidomain enzyme with several distinct structural and functional regions, heterogeneity in residue mobility was evident. Special attention was paid to residues within and near the active site. Residues that formed non-bonded interactions with the ligands mainly those belonging to domains A and B showed low fluctuations, reflecting structural rigidity and the potential to maintain steady ligand contacts. Conversely, flexible loop elements, such as the B domain loop and the active-site lid, exhibited greater mobility. The reduced flexibility of residues at the binding site underlines their importance in preserving stable interactions with both ligands throughout the simulations. In addition, RMSF values of the heavy atoms of acarbose and compound 13j were determined and are presented in Fig. 9. Both ligands showed very limited fluctuations, with all values remaining below 0.25 nm. The least mobile atoms in both ligands were those embedded in ring systems, whose intrinsic rigidity restricts flexibility. This rigidity likely enhances the ability of ring atoms to form stable non-bonded interactions with residues in the binding pocket. At the same time, the establishment of these strong contacts further constrains the motion of ring atoms, reinforcing the reciprocal stability between the ligands and the enzyme's active site.

### Prediction of pharmacokinetics

Pharmacokinetics of the best potent compound 13j in the present project was evaluated *in silico* in terms of three aspects: druglikeness, ADME (absorption, distribution, metabolism, and excretion), and toxicity. All the obtained results were compared with those of acarbose, an approved α-glucosidase inhibitor. For this propose, we used four credible online software tools: SwissADME, pkCSM, PreADMET, and admetSAR.^33–36^ The obtained data of the SwissADME, pkCSM, and PreADMET, were listed in Table 2.

**Table 2:** Prediction of the pharmacokinetics of compound 13j as the most potent compound and acarbose as the standard drug

Pharmacokinetics		Compound 13j	Acarbose
Druglikeness	Rule of five	Violated	Violated
	Veber rule	Violated	Violated
ADME	GI absorption	Low	Low
	BBB permeant	No	No
	P-gp substrate	No	Yes
	CYP1A2 inhibitor	Yes	No
	CYP2C19 inhibitor	Yes	No
	CYP2C9 inhibitor	Yes	No
	CYP2D6 inhibitor	No	No
	CYP3A4 inhibitor	No	No
	Total clearance	−0.127	0.702
	OCT2 substrate	No	No
Toxicity	Ames_test	Non-mutagen	Mutagen
	Carcino_Mouse	Positive	Positive
	Carcino_Rat	Negative	Negative
	hERG_inhibition	Low risk	Ambiguous

Druglikeness calculations for compound 13j and acarbose showed that neither of them complies with the Lipinski rule (rule of five) or the Veber rule, which are two important rules for predicting druglikeness.

Absorption predictions indicated that both compound 13j and acarbose have not gastrointestinal absorption (GI) and permeability to blood–brain barrier (BBB). Moreover, distribution predictions showed that compound 13j is not a substrate for P-glycoprotein (P-gp, an efflux pump) whereas acarbos is a P-gp substrate.^37^ In term of metabolism, compound 13j properly inhibits CYP1A2, CYP2C19, and CYP2C9 isoforms of cytochrome P450 (CYP), a major drug-metabolizing enzyme, and dose not inhibit CYP2D6 and CYP3A4 isoforms but acarbose does not inhibit any of these enzymes. According to Table 2, in terms of excretion, total clearance of compound 13j is lower than acarbose. Moreover, compound 13j and acarbose are not substrates for organic cation transporter 2 (OCT2).

Toxicity prediction of acarbose and compound 13j indicated that compound 13j is non-mutagenic, whereas computational analysis suggested a potential mutagenic alert for acarbose. Nevertheless, it should be emphasized that this result is obtained from *in silico* predictions and may not reflect the well-documented clinical safety of acarbose reported in the literature. In terms of carcinogenicity, compounds 13j and acarbose show similar profile. Finally, the potential of cardiotoxicity (hERG inhibition) of compound 13j is low, whereas this type of toxicity for acarbose remains ambiguous.

**Fig. 10:**
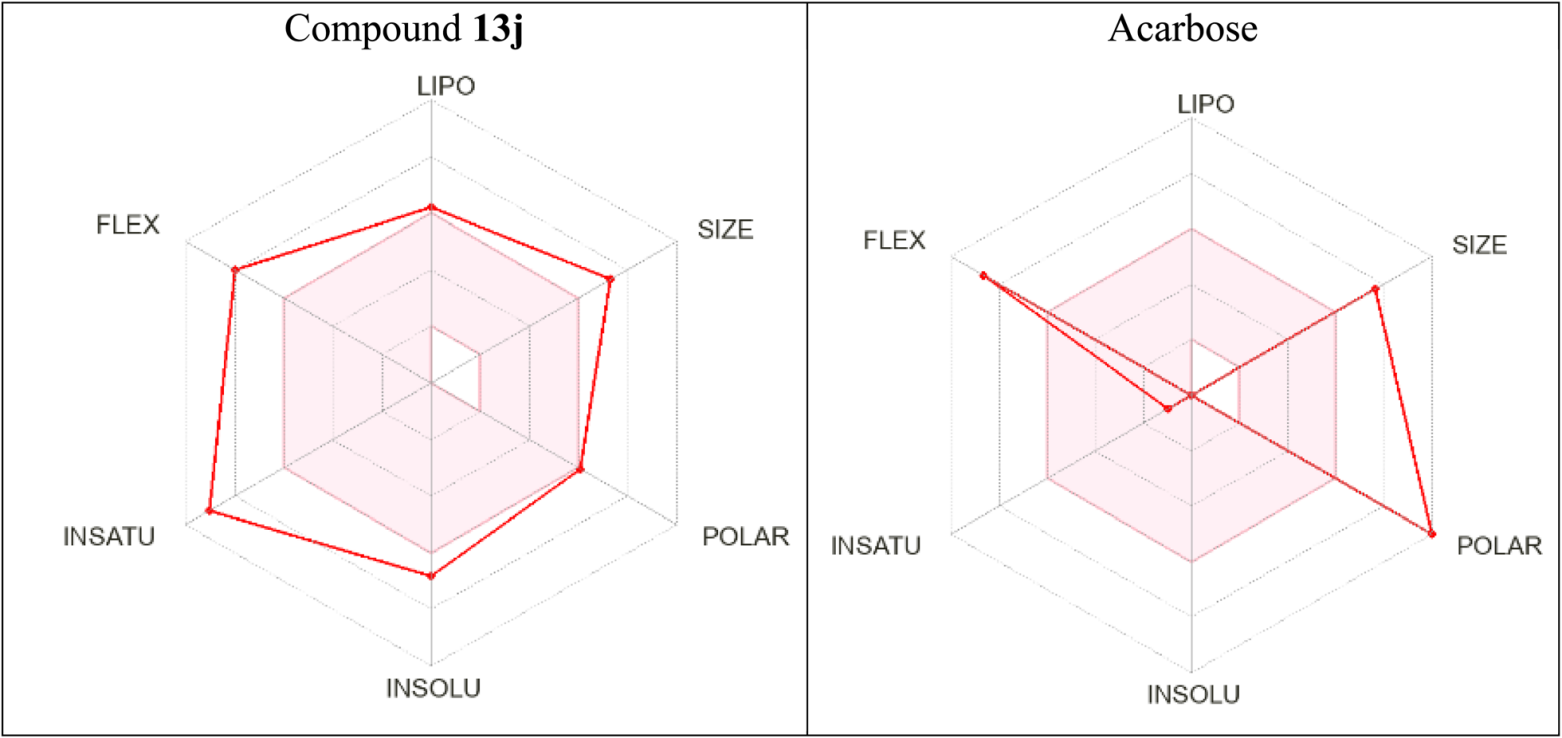
Bioavailability radars of compounds 13j and acarbose.

Bioavailability radars of the new compound 13j and the standard inhibitor (acarbose) are shown in Fig. 10. In these radars, the pink area demonstrated a suitable physicochemical space for oral bioavailability. According to the obtained bioavailability radars, compound 13j does not fall within the pink area whereas acarbose is positioned in this area only marginally.

**Fig. 11:**
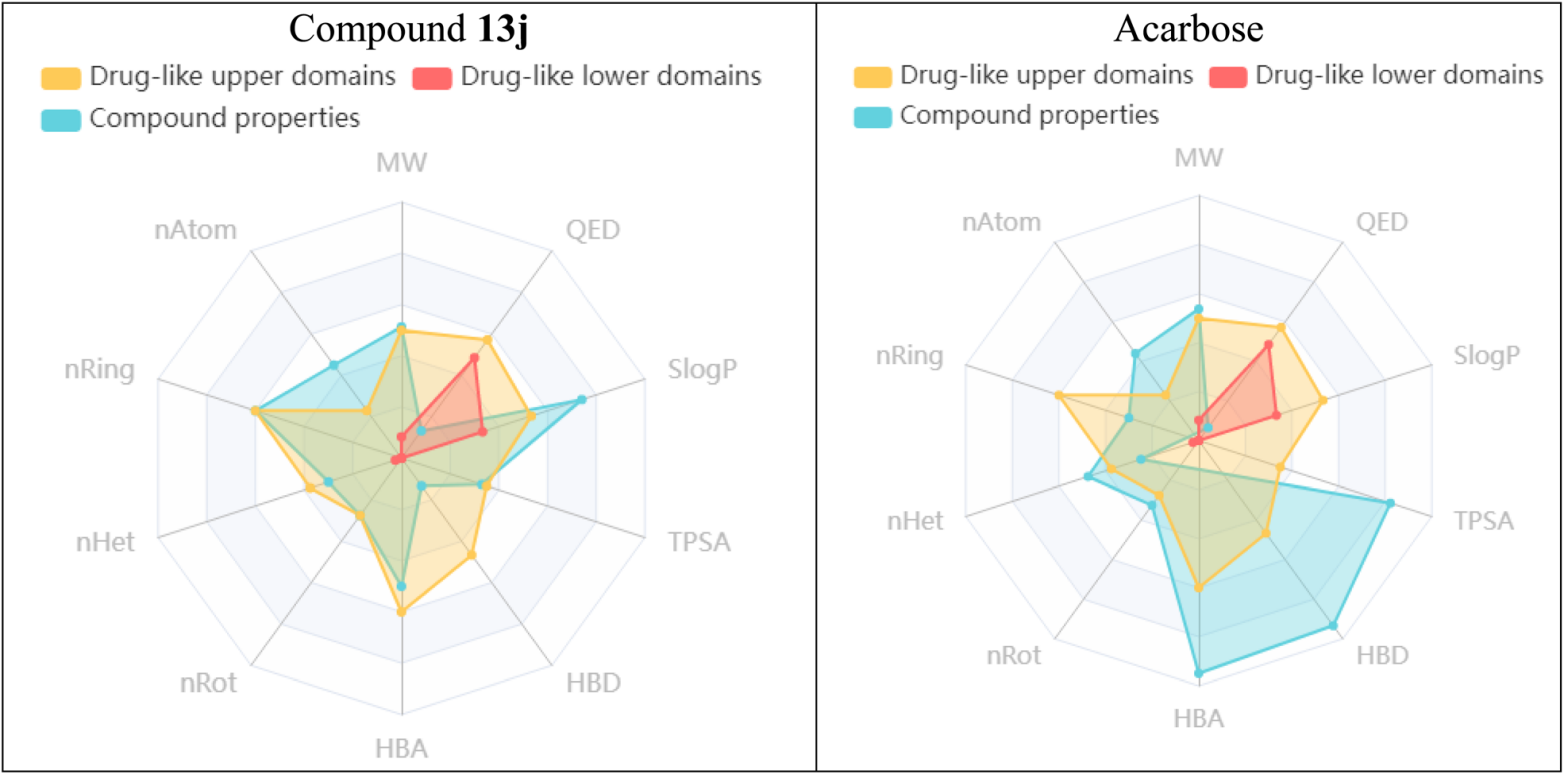
The predicted ADMET profiles of compound 13j and acarbose using the admetSAR server.

Using the admetSAR server, the ADMET properties of compound 13j were also predicted and compared with those of the acarbose. The obtained data of the admetSAR were illustrated in Fig. 11. According to this figure, compound 13j, shows a higher presence within the drug-likeness acceptable region compared to acarbose. This compound has favorable pharmacokinetic characteristics, including improved intestinal absorption and oral bioavailability in comparison to acarbose, while maintaining an acceptable toxicity profile.

## Conclusion

Fourteen new 5,6-diphenyl-1,2,4-triazine-hydrazineylidene-phenoxy-1,2,3-triazole-acetamide derivatives 13a–n were successfully synthesized as potent anti-α-glucosidase agents. These compounds were assessed against the latter enzyme. All of the synthesized 1,2,4-triazine derivatives exhibited significant inhibitory activity against α-glucosidase when compared with acarbose as a standard drug. Among compound 13a–n, compound 13j, with inhibitory activity 6250 times more than that of acarbose, was identified as the most potent α-glucosidase inhibitor. Moreover, comparison of the new title compounds 13a–n with the templates used in their design showed that our new compounds were significantly more potent than the templates. Therefore, we can claim that we have introduced valuable lead compounds for the development of new potent α-glucosidase inhibitors. *In vitro* kinetic study on compound 13j revealed that it acts as an uncompetitive inhibitor, whereas acarbose is a competitive inhibitor. Prediction of drug-likness, ADME, and toxicity for compound 13j and acrabose demonstrated that these compounds are approximately similar in terms of these pharmacokinetics parameters.

### Limitations of the study

While this study presents compound 13j as a potent inhibitor of yeast α-glucosidase, several limitations should be acknowledged. Firstly, the inhibitory activity was evaluated using an enzyme from a yeast, which may not fully replicate the kinetics or physiological relevance of the human α-glucosidase. Secondly, and most importantly, no *in vivo* anti-diabetic efficacy studies were conducted on compound 13j. Finally, safety profile of this compound requires validation through extensive *in vivo* toxicological studies in an animal model. Therefore, the compound should currently be regarded as a promising *in vitro* lead, and its development into a therapeutic agent hinges on future *in vivo* investigations.

## Author contributions

MM and SM conceived, designed, and supervised this study. NA synthesized and interpreted analytical data of compounds. *In vitro* experiments were performed by MAF. *In silico* studies were performed and written by MM-K. The manuscript was drafted by MM-K and NA. MM reviewed and edited the drafts.

## Conflicts of interest

The authors declare no conflict of interest.

## Supplementary Material

RA-015-D5RA06909B-s001

## Data Availability

The data supporting this article have been included as part of the supplementary information (SI). Supplementary information is available. See DOI: https://doi.org/10.1039/d5ra06909b.
