# Supplementary material for: New 5,6-diphenyl-1,2,4-triazine-hydrazineylidene-phenoxy-1,2,3-triazole-acetamide derivatives as potent synthetic α-glucosidase inhibitors
Source: RSC Adv. 2025 Oct 15;15(46):38547–61. doi: 10.1039/d5ra06909b (PMC12523282; doi:10.1039/d5ra06909b)

Supplementary Information (SI) for RSC Advances.
This journal is © The Royal Society of Chemistry 2025

Support information

New 5,6-diphenyl-1,2,4-triazine-hydrazineylidene-phenoxy-1,2,3-triazole-acetamide derivatives as the potent synthetic α-glucosidase inhibitors

Nafise Asemanipoor[1], Shahram Moradi[1]*, Mohammad Ali Faramarzi[2], Maryam Mohammadi-Khanaposhtani[3], Mohammad Mahdavi[4]*

[1] Department of Chemistry, NT.C., Islamic Azad University, Tehran, Iran

[2] Department of Pharmaceutical Biotechnology, Faculty of Pharmacy and Biotechnology Research Center, Tehran University of Medical Sciences, Tehran, Iran

[3] Cellular and Molecular Biology Research Center, Health Research Institute, Babol University of Medical Sciences, Babol, Iran

[4] Endocrinology and Metabolism Research Center, Endocrinology and Metabolism Clinical Sciences Institute, Tehran University of Medical Sciences, Tehran, Iran.

*Corresponding authors.

E-mail addresses: shm_moradi@yahoo.com (S. Moradi), momahdavi@tums.ac.ir (M. Mahdavi).

(E)-2-(4-((4-((2-(5,6-diphenyl-1,2,4-triazin-3-yl)hydrazineylidene)methyl)phenoxy)methyl)-1H-1,2,3-triazol-1-yl)-N-phenylacetamide (**13a**)

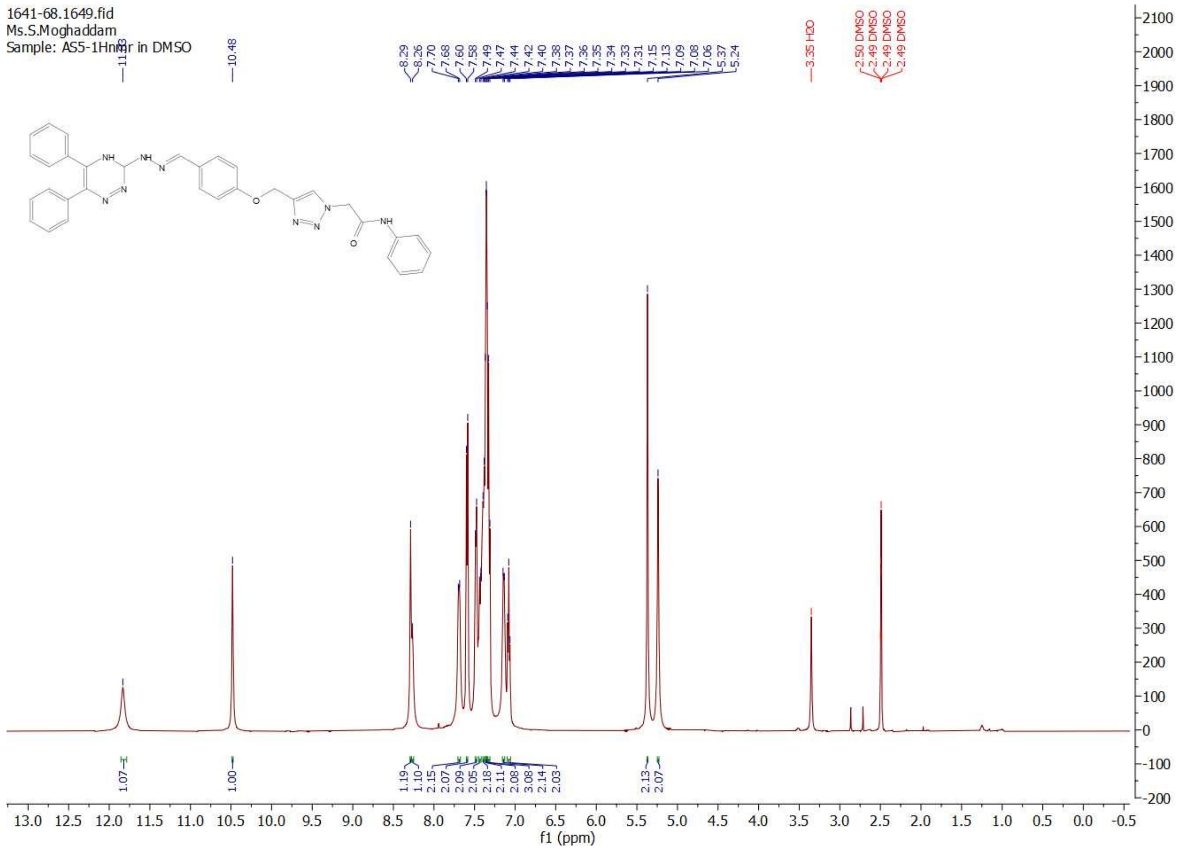

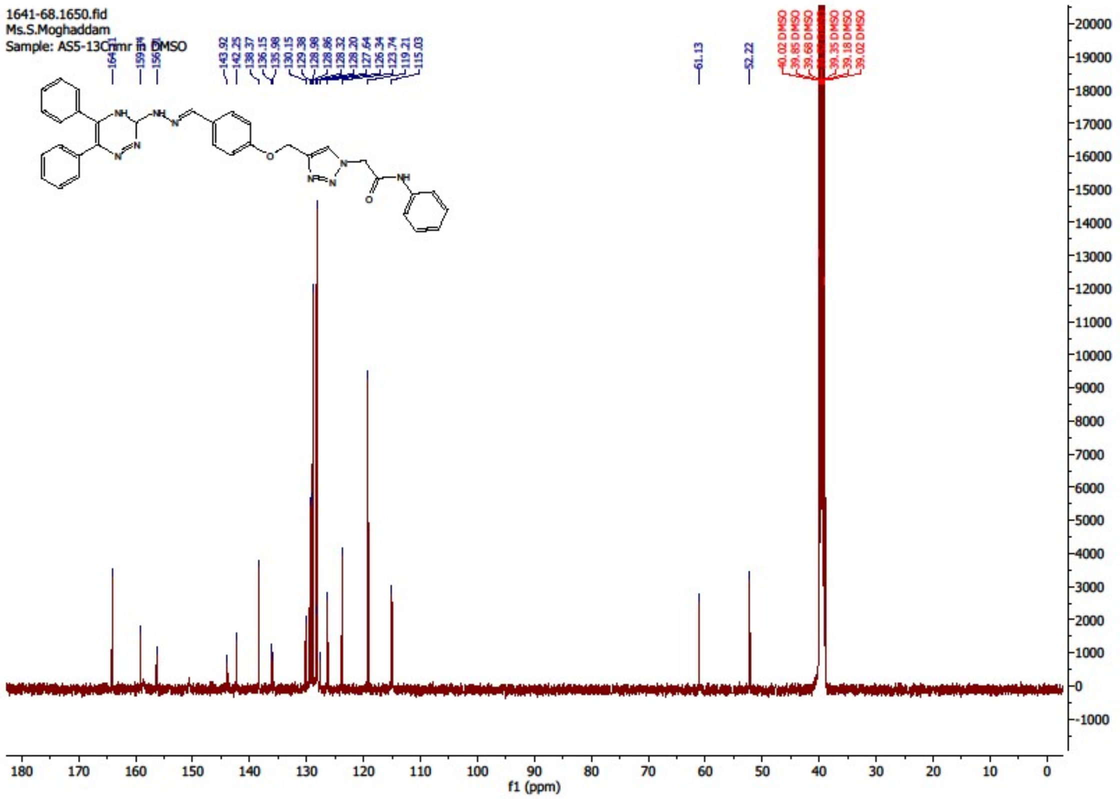

(E)-2-(4-((4-((2-(5,6-diphenyl-1,2,4-triazin-3-yl)hydrazineylidene)methyl)phenoxy)methyl)-1H-1,2,3-triazol-1-yl)-N-(o-tolyl)acetamide (**13b**)

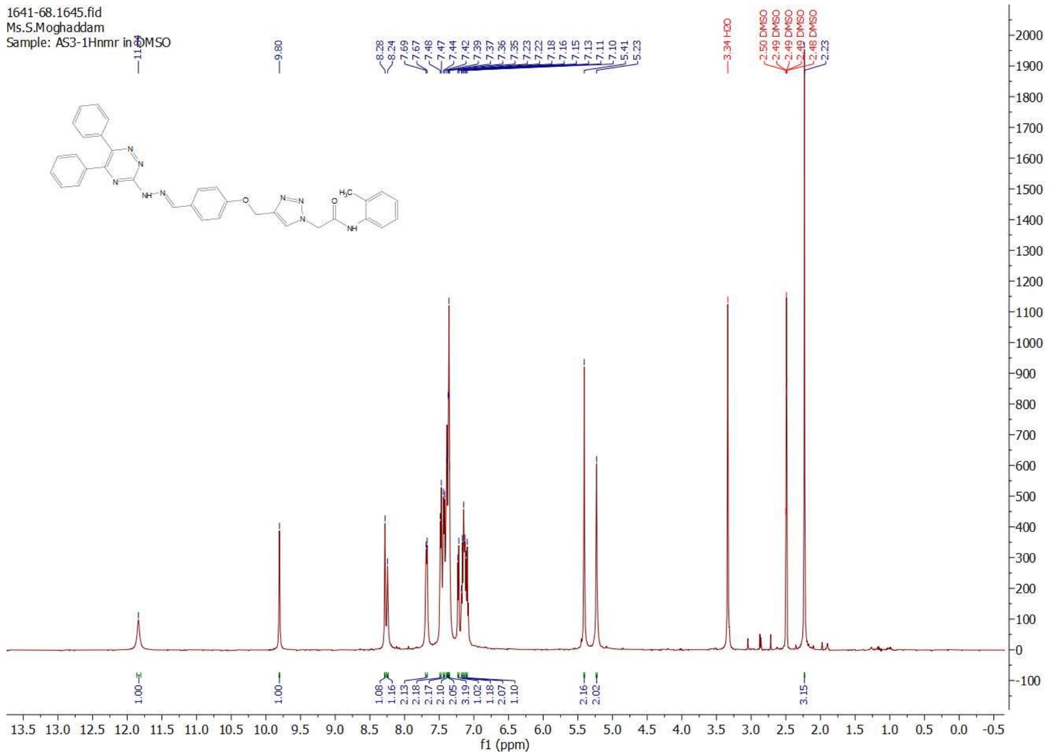

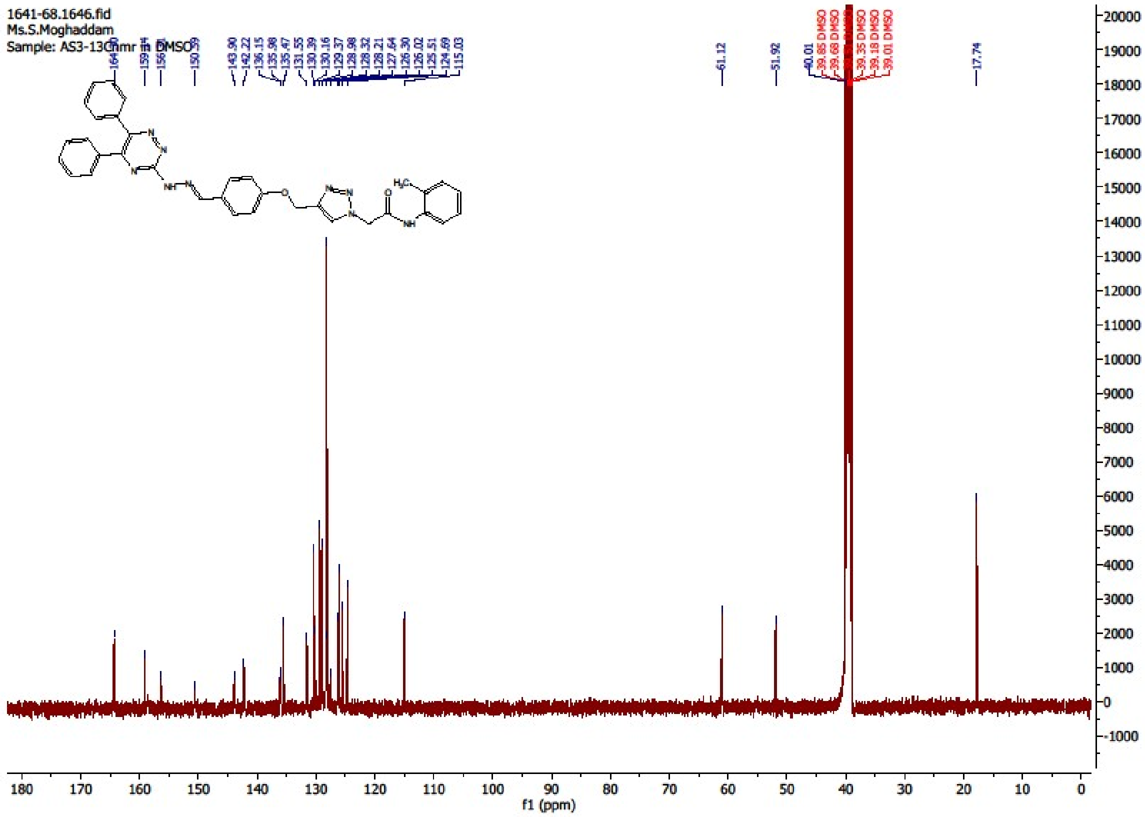

(E)-2-(4-((4-((2-(5,6-diphenyl-1,2,4-triazin-3-yl)hydrazineylidene)methyl)phenoxy)methyl)-1H-1,2,3-triazol-1-yl)-N-(m-tolyl)acetamide (**13c**)

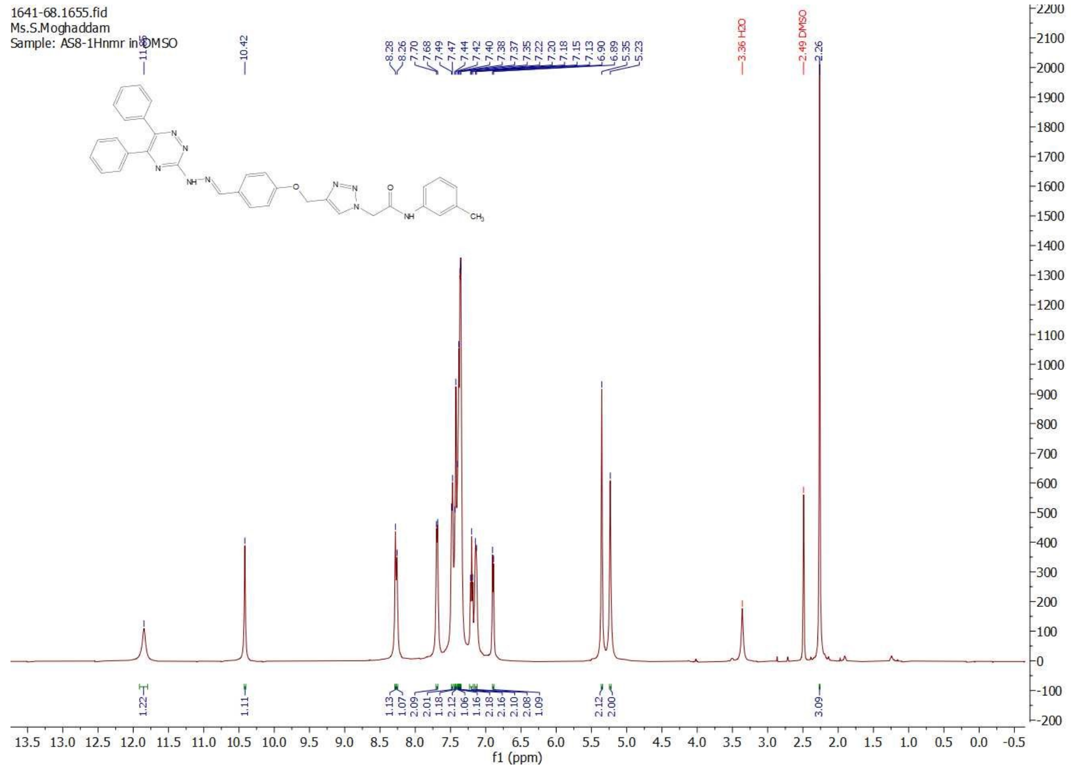

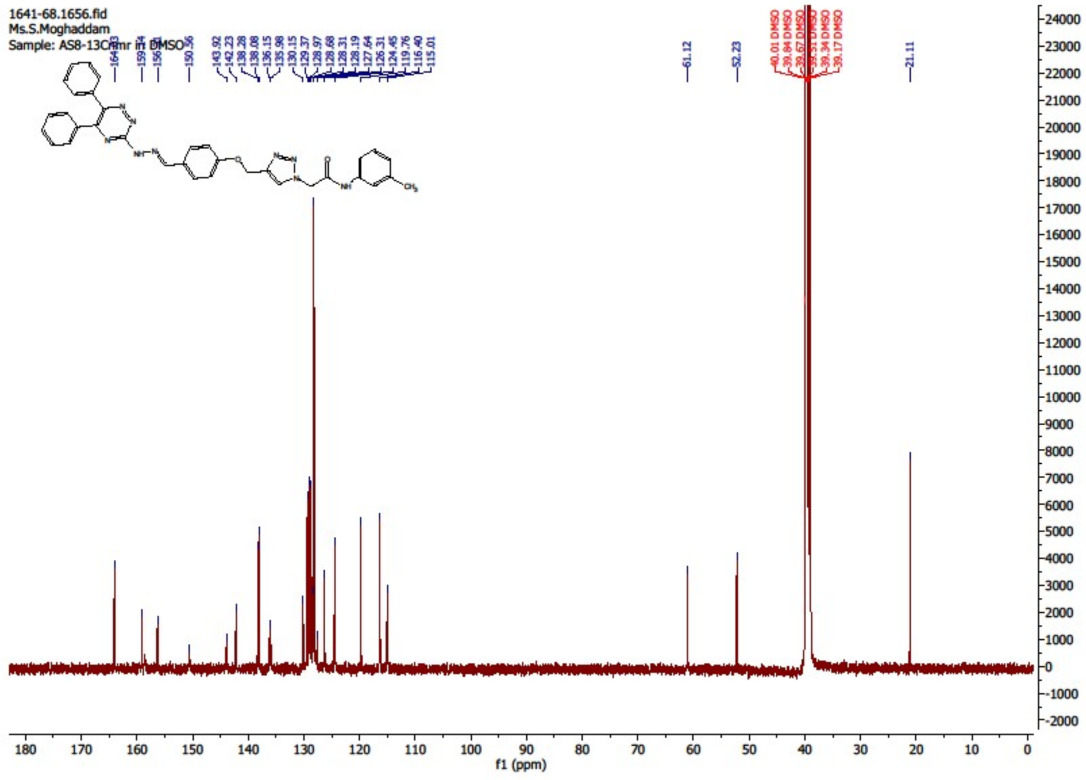

(E)-N-(2,4-dimethylphenyl)-2-(4-((4-((2-(5,6-diphenyl-1,2,4-triazin-3-yl)hydrazineylidene)methyl)phenoxy)methyl)-1H-1,2,3-triazol-1-yl)acetamide (**13d**)

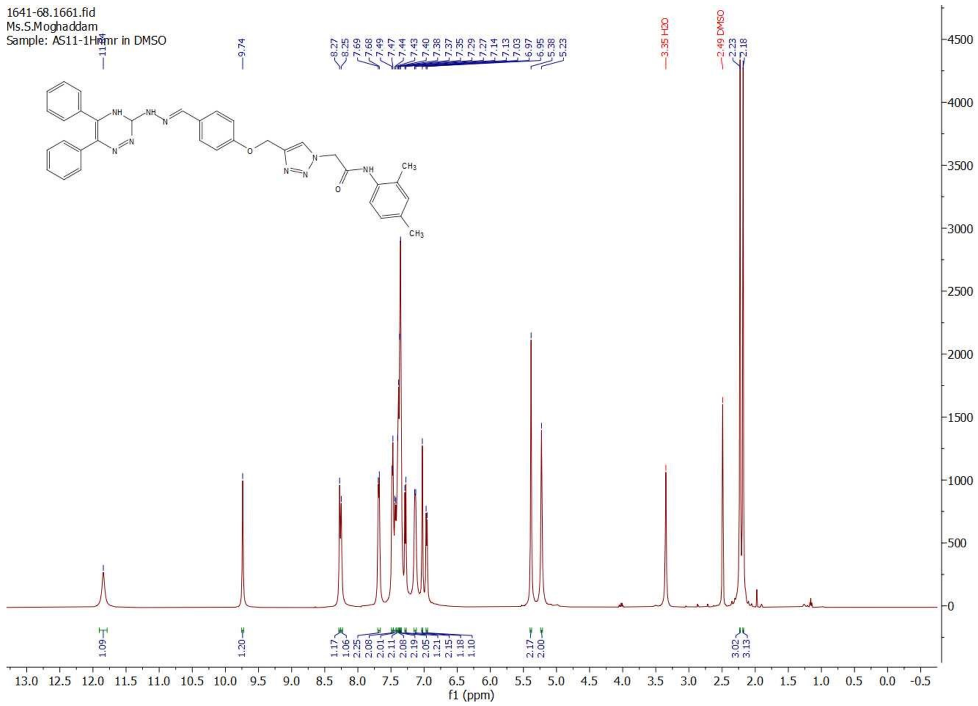

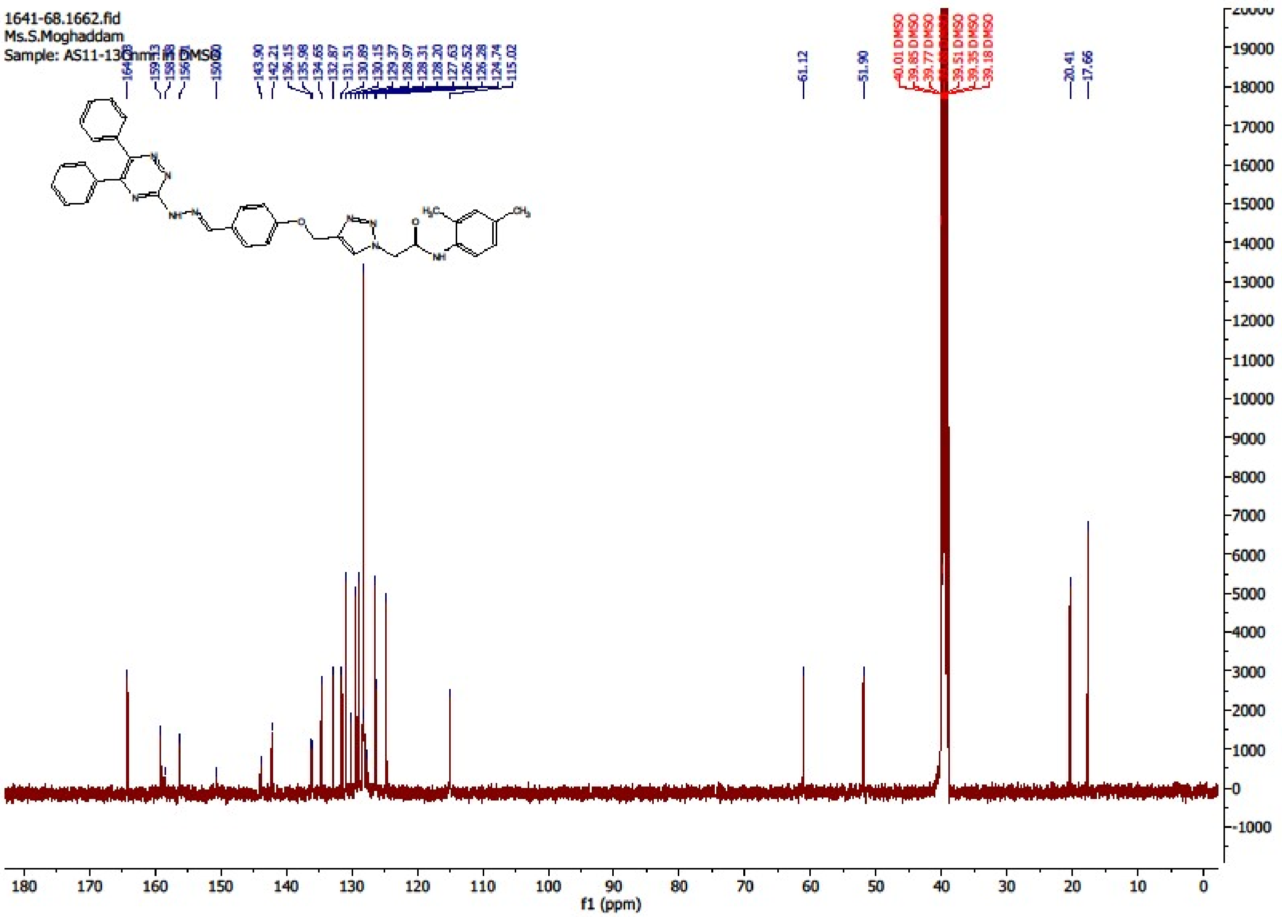

(E)-2-(4-((4-((2-(5,6-diphenyl-1,2,4-triazin-3-yl)hydrazineylidene)methyl)phenoxy)methyl)-1H-1,2,3-triazol-1-yl)-N-(4-ethylphenyl)acetamide (**13e**)

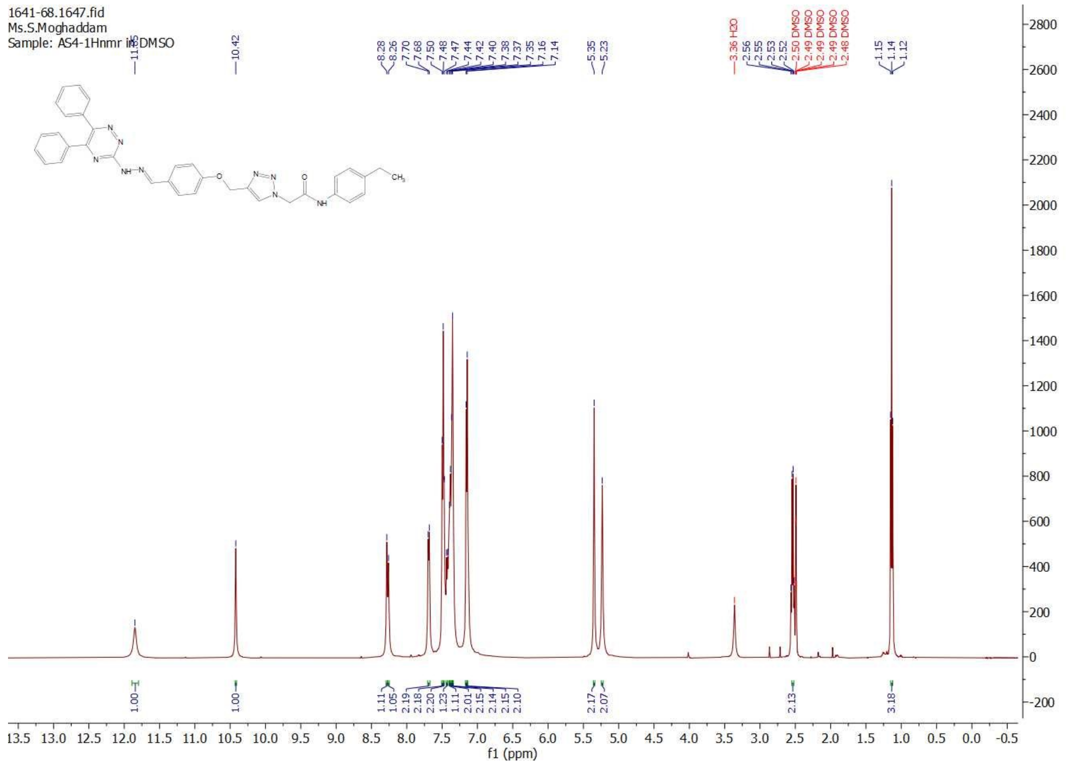

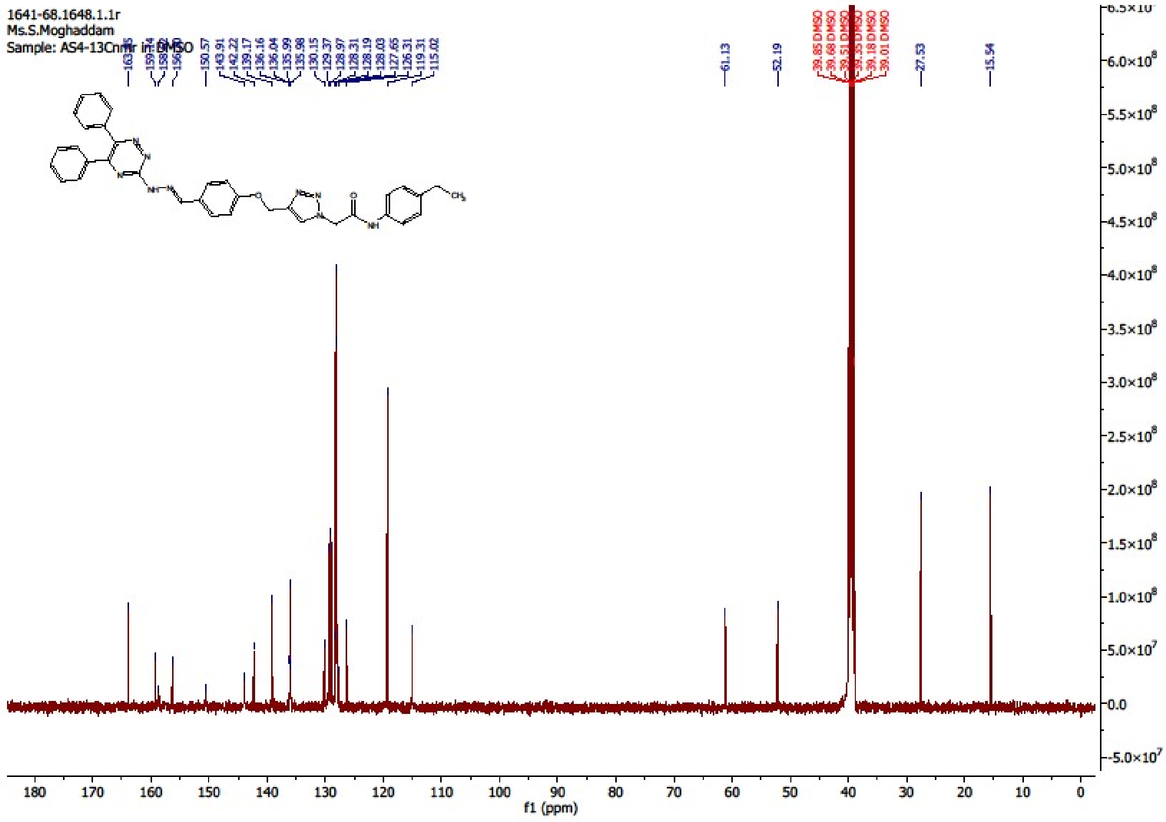

(E)-2-(4-((4-((2-(5,6-diphenyl-1,2,4-triazin-3-yl)hydrazineylidene)methyl)phenoxy)methyl)-1H-1,2,3-triazol-1-yl)-N-(4-methoxyphenyl)acetamide (**13f**)

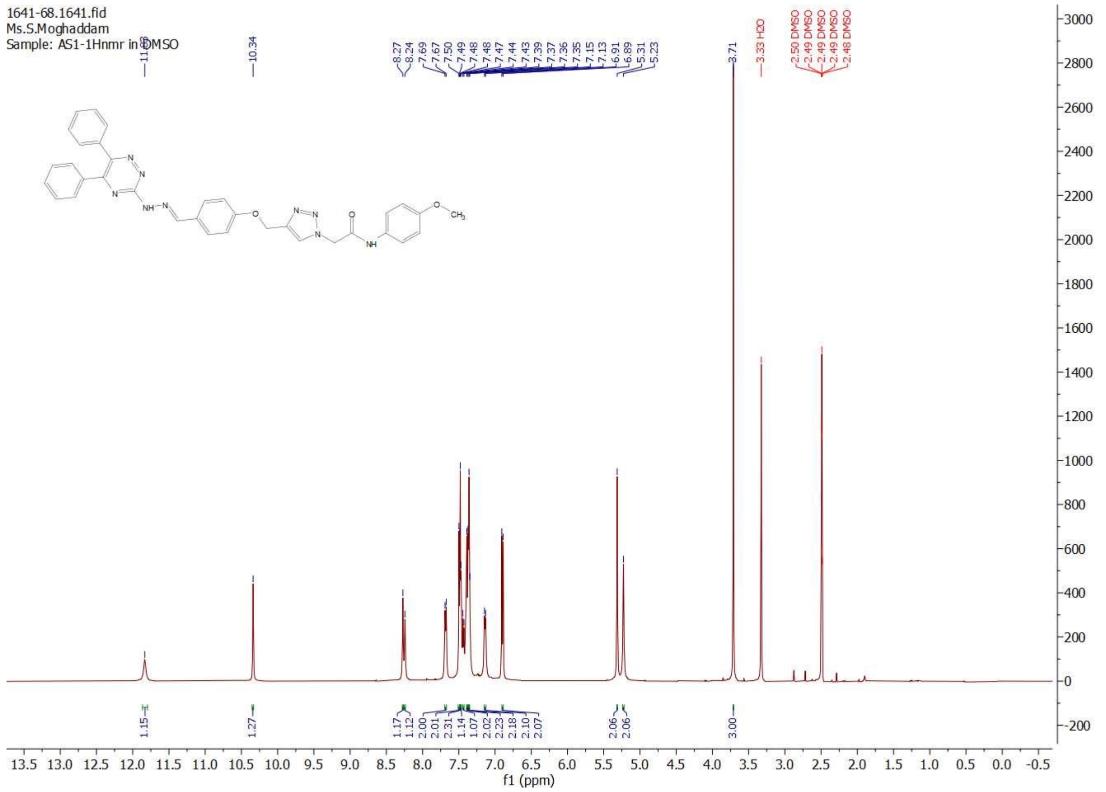

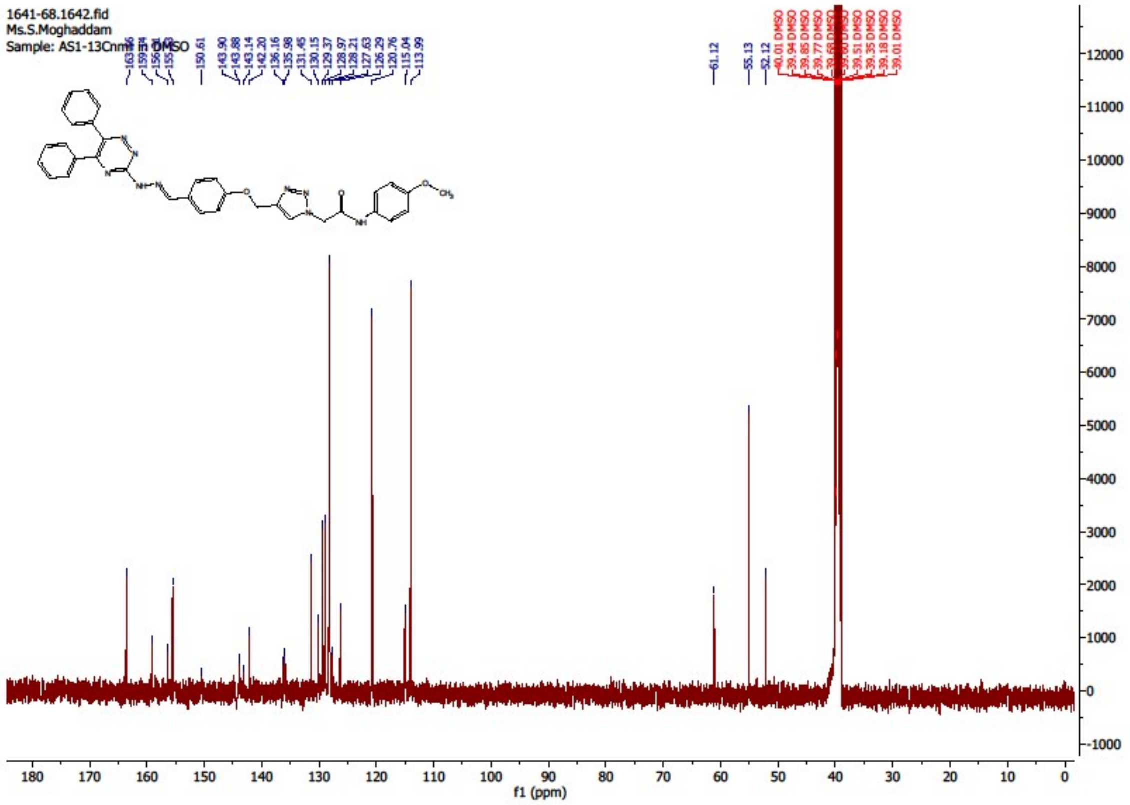

(E)-2-(4-((4-((2-(5,6-diphenyl-1,2,4-triazin-3-yl)hydrazineylidene)methyl)phenoxy)methyl)-1H-1,2,3-triazol-1-yl)-N-(2-fluorophenyl)acetamide (**13g**)

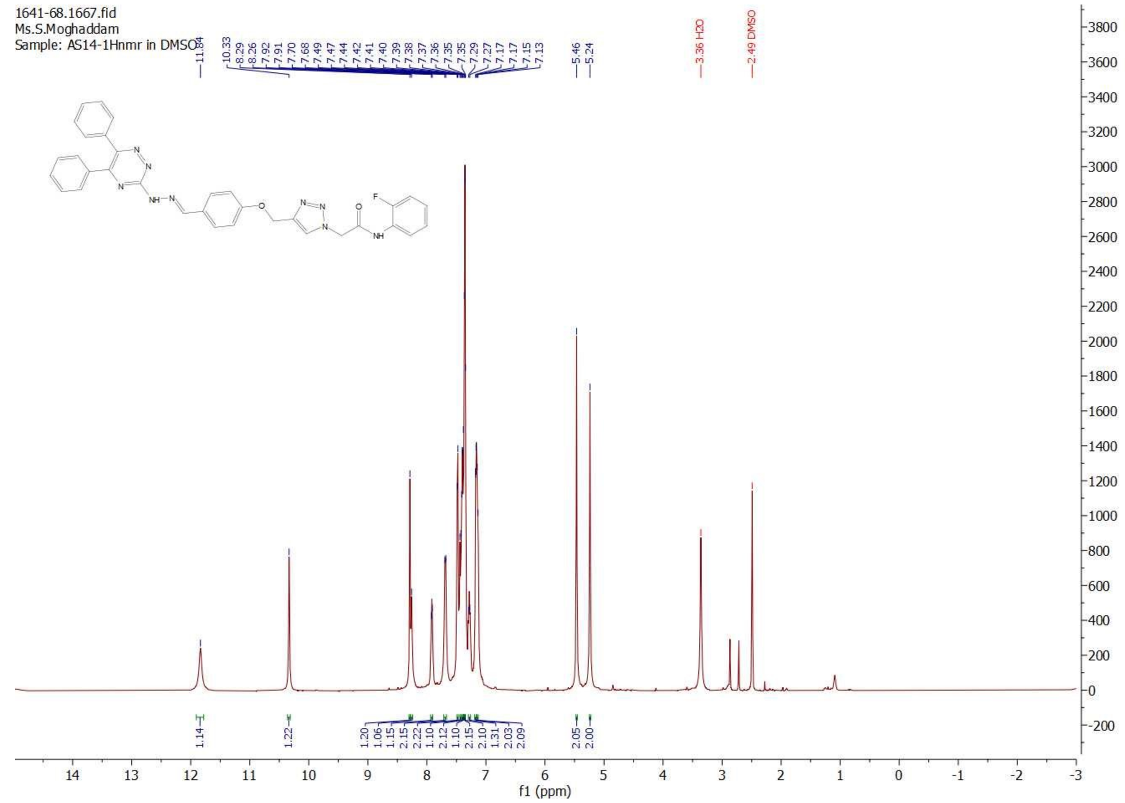

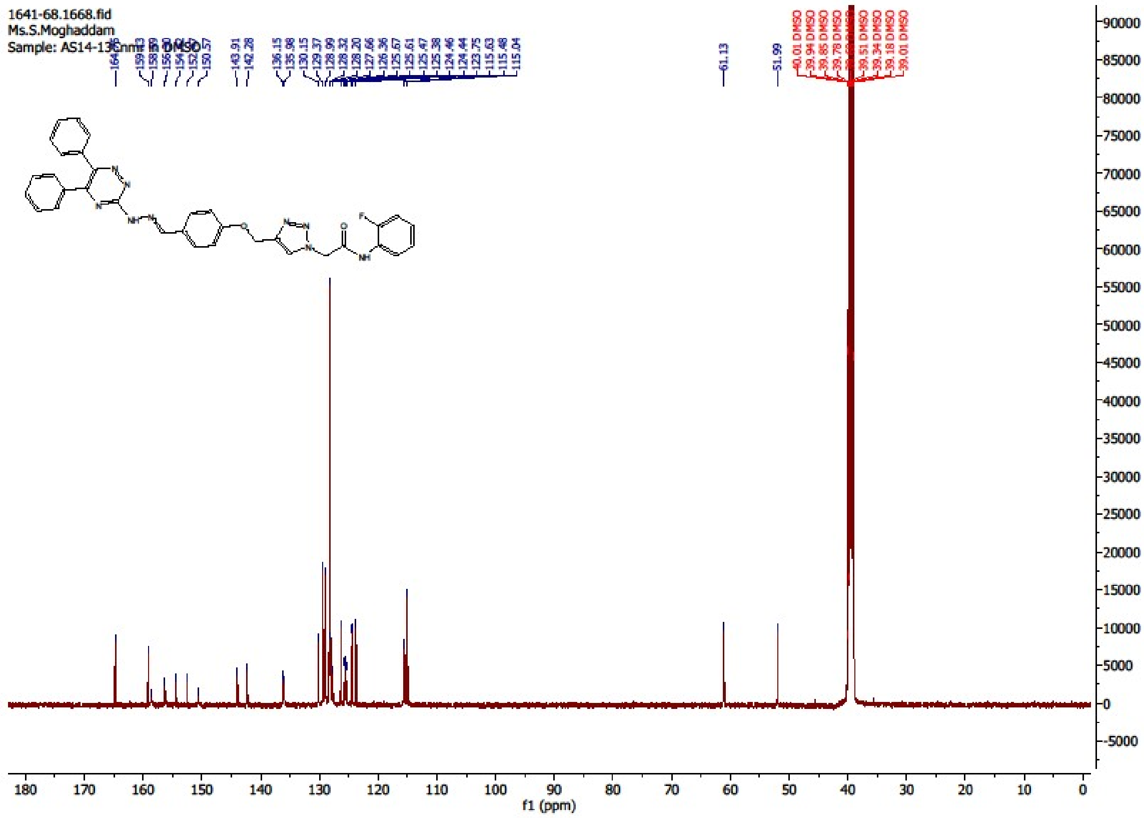

(E)-N-(2-chlorophenyl)-2-(4-((4-((2-(5,6-diphenyl-1,2,4-triazin-3-yl)hydrazineylidene)methyl)phenoxy)methyl)-1H-1,2,3-triazol-1-yl)acetamide (**13h**)

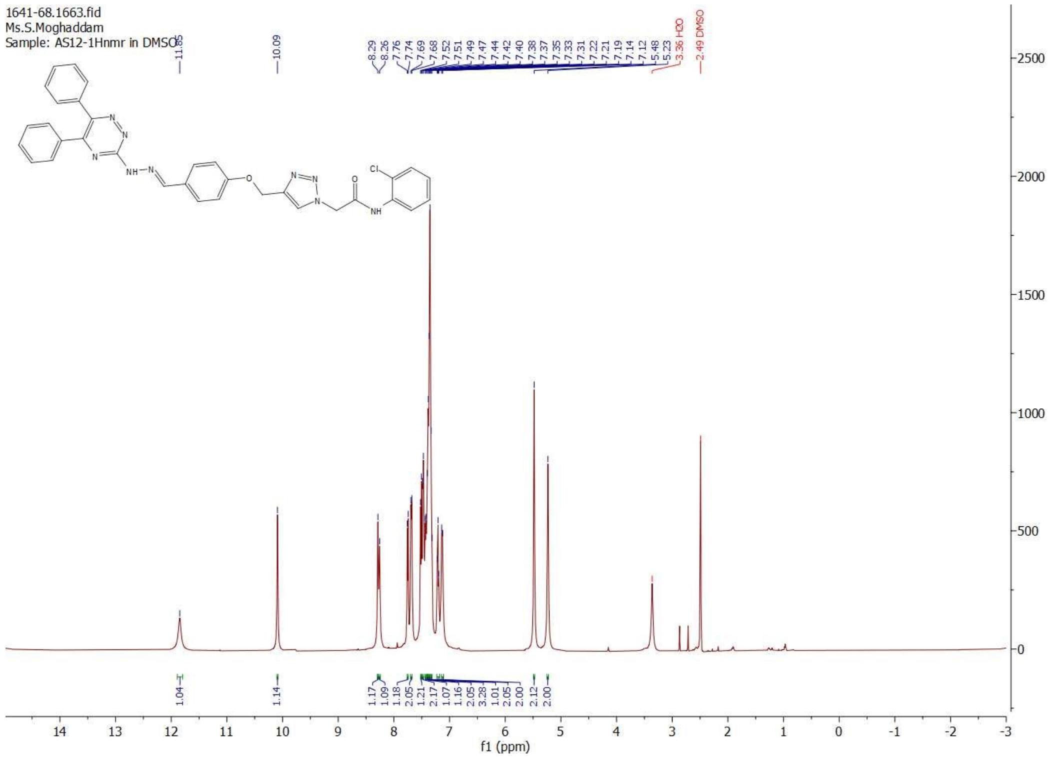

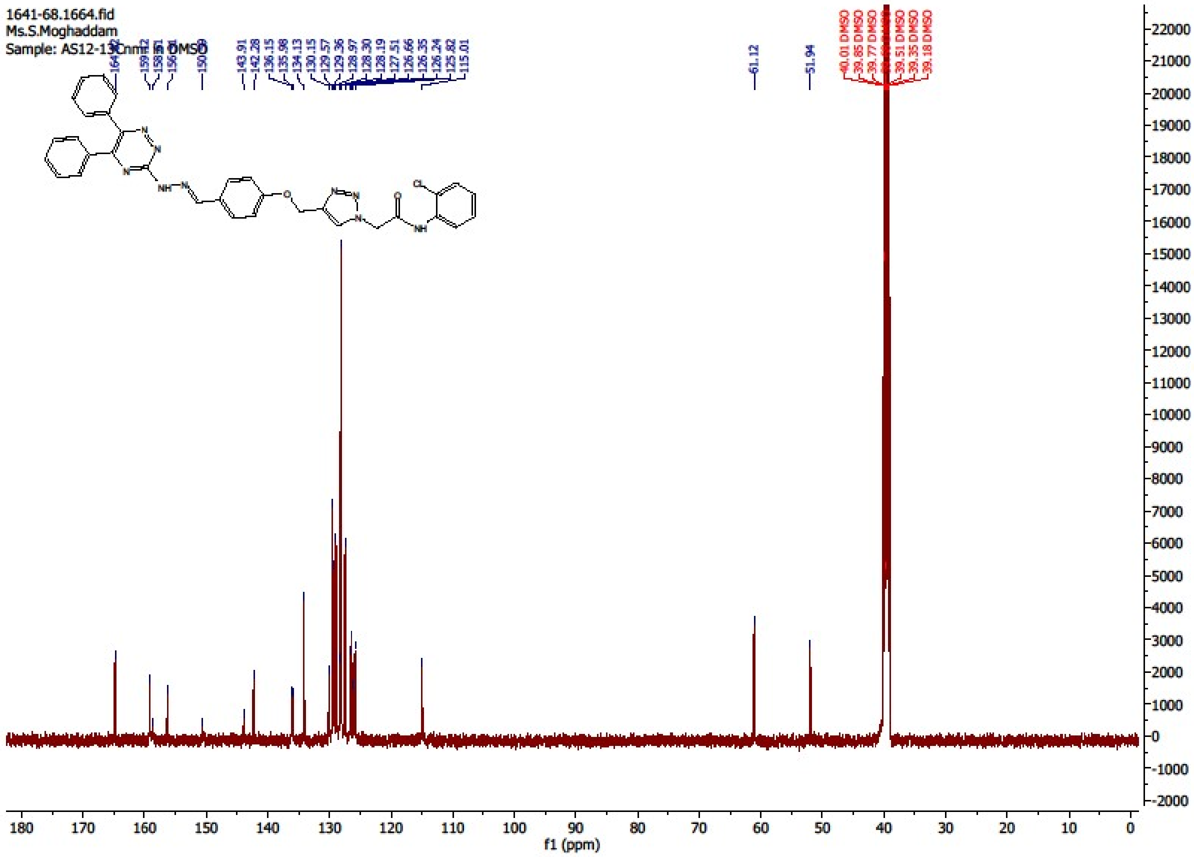

(E)-N-(3-chlorophenyl)-2-(4-((4-((2-(5,6-diphenyl-1,2,4-triazin-3-yl)hydrazineylidene)methyl)phenoxy)methyl)-1H-1,2,3-triazol-1-yl)acetamide (**13i**)

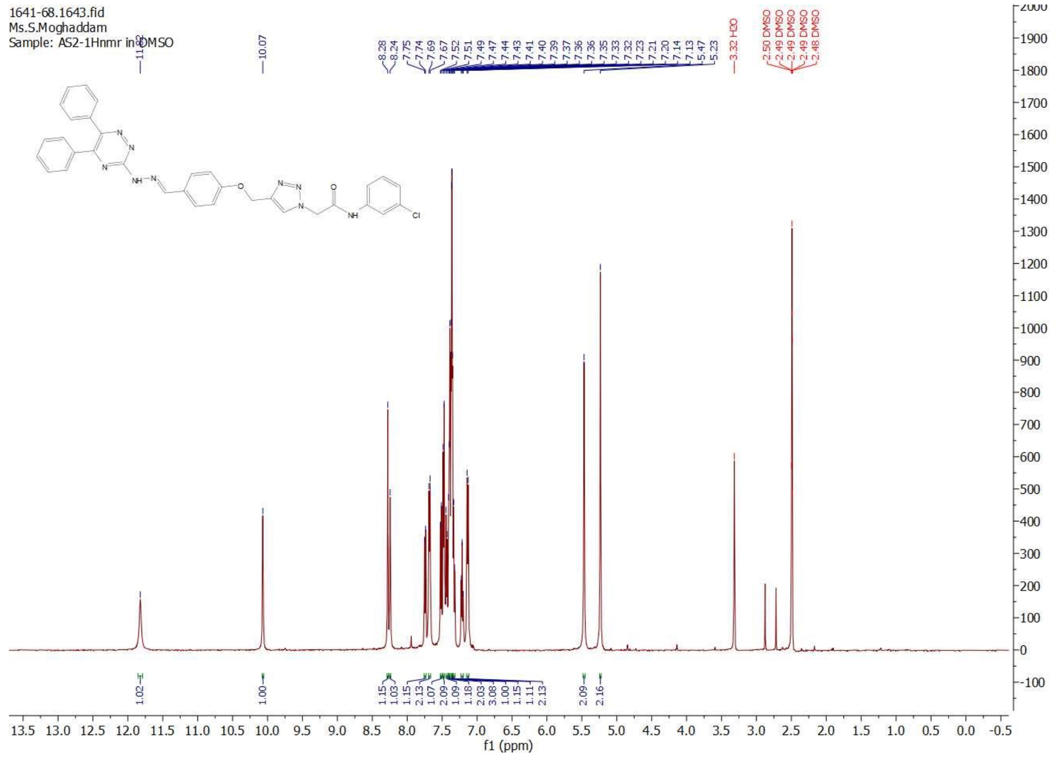

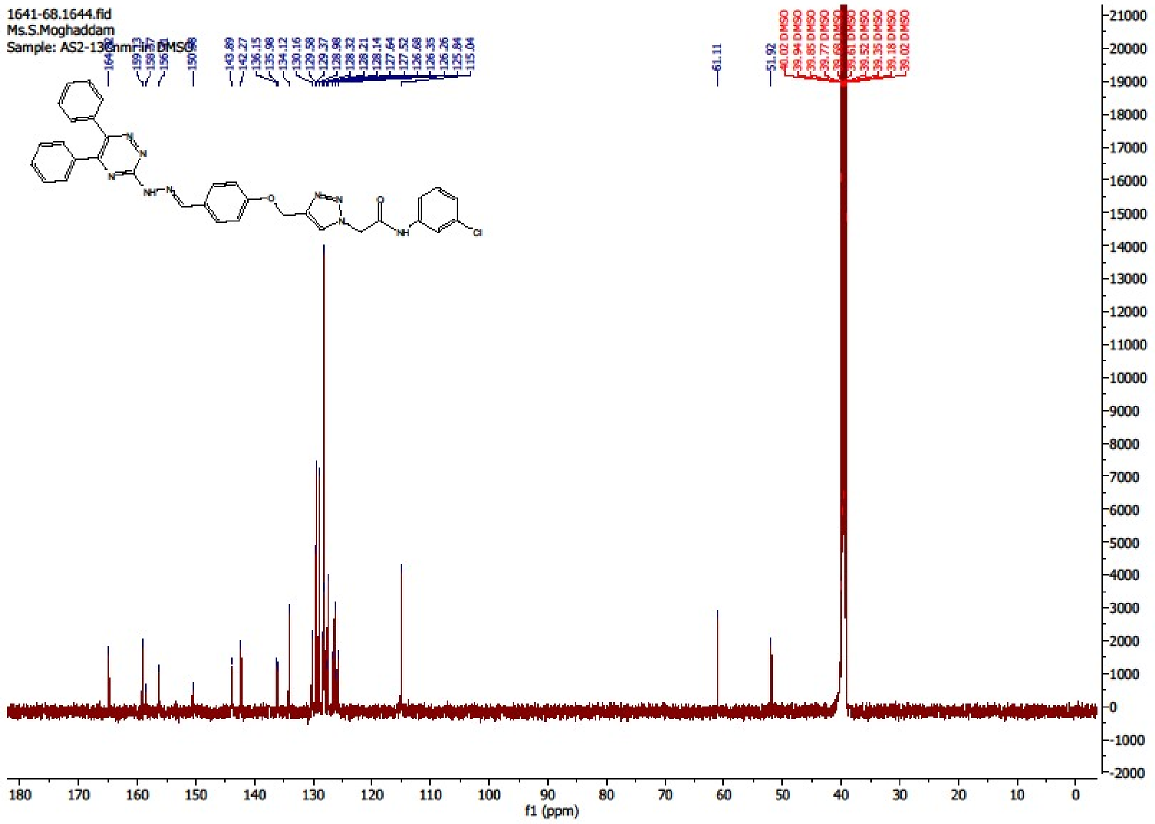

(E)-N-(4-chlorophenyl)-2-(4-((4-((2-(5,6-diphenyl-1,2,4-triazin-3-yl)hydrazineylidene)methyl)phenoxy)methyl)-1H-1,2,3-triazol-1-yl)acetamide (**13j**)

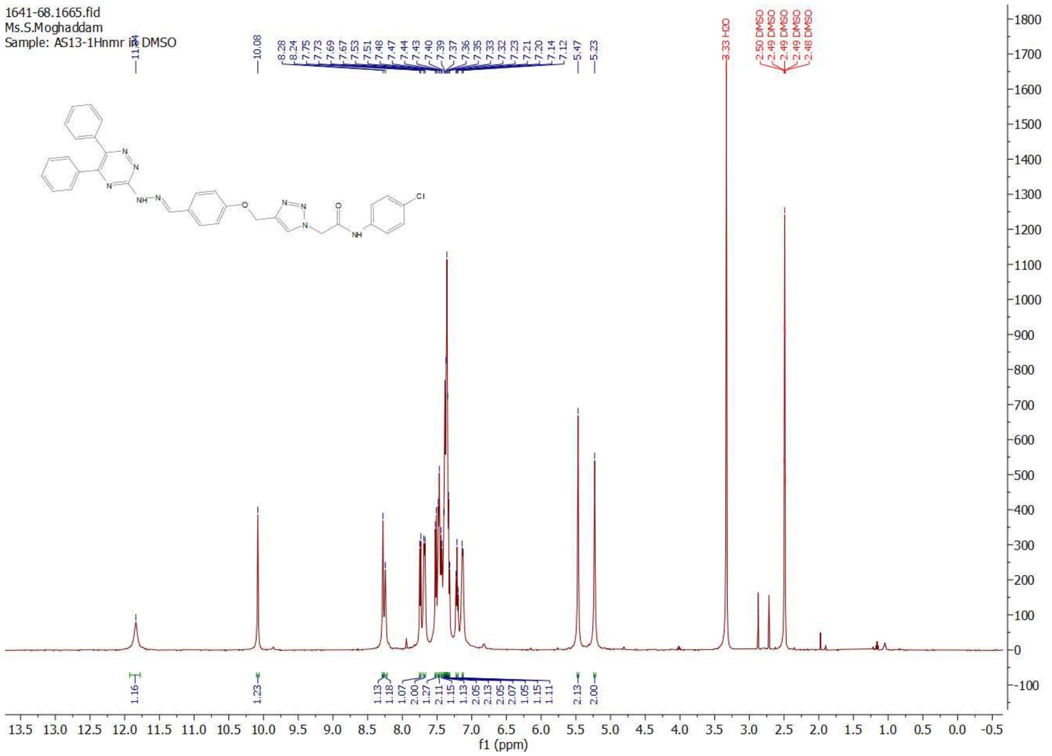

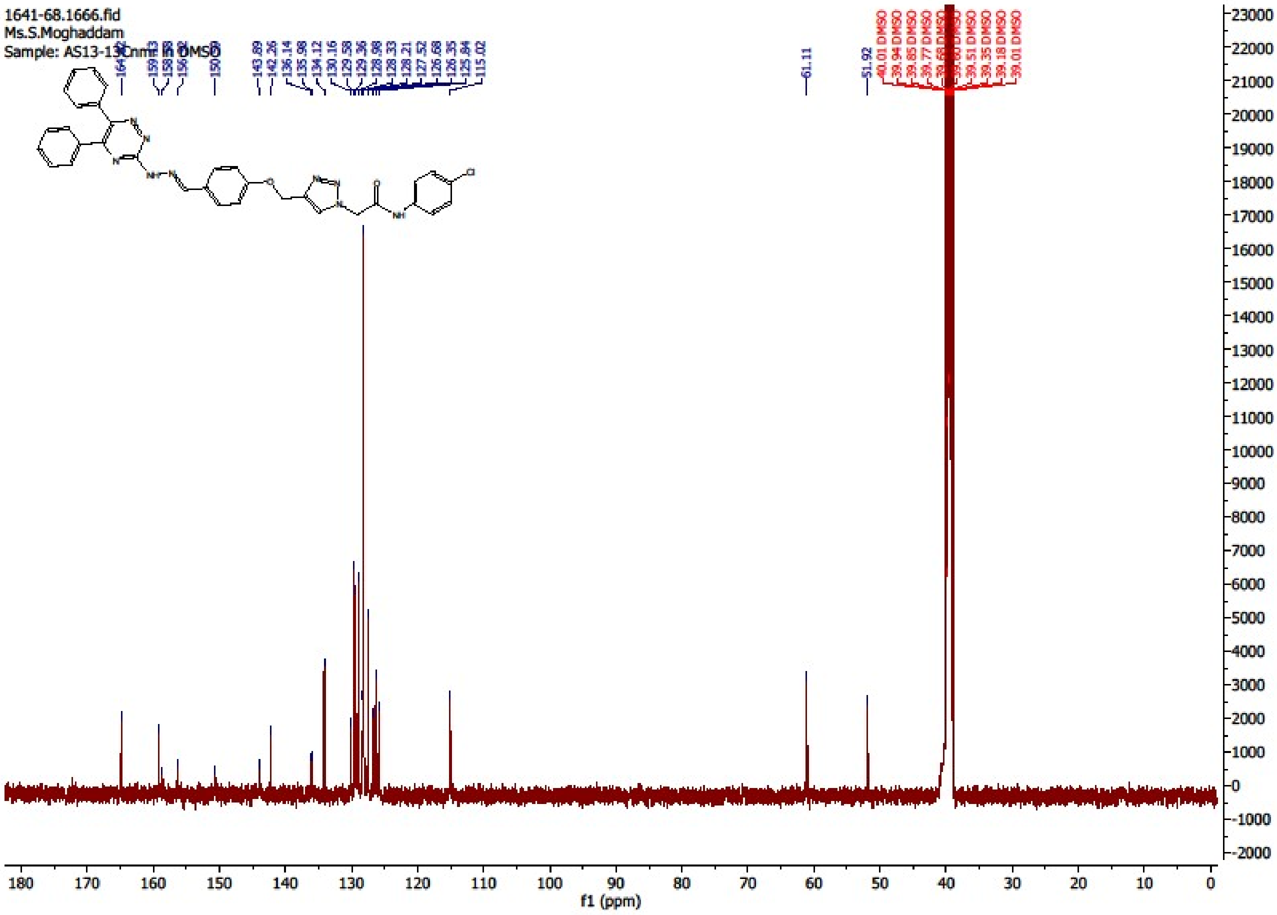

(E)-N-(4-bromophenyl)-2-(4-((4-((2-(5,6-diphenyl-1,2,4-triazin-3-yl)hydrazineylidene)methyl)phenoxy)methyl)-1H-1,2,3-triazol-1-yl)acetamide (**13k**)

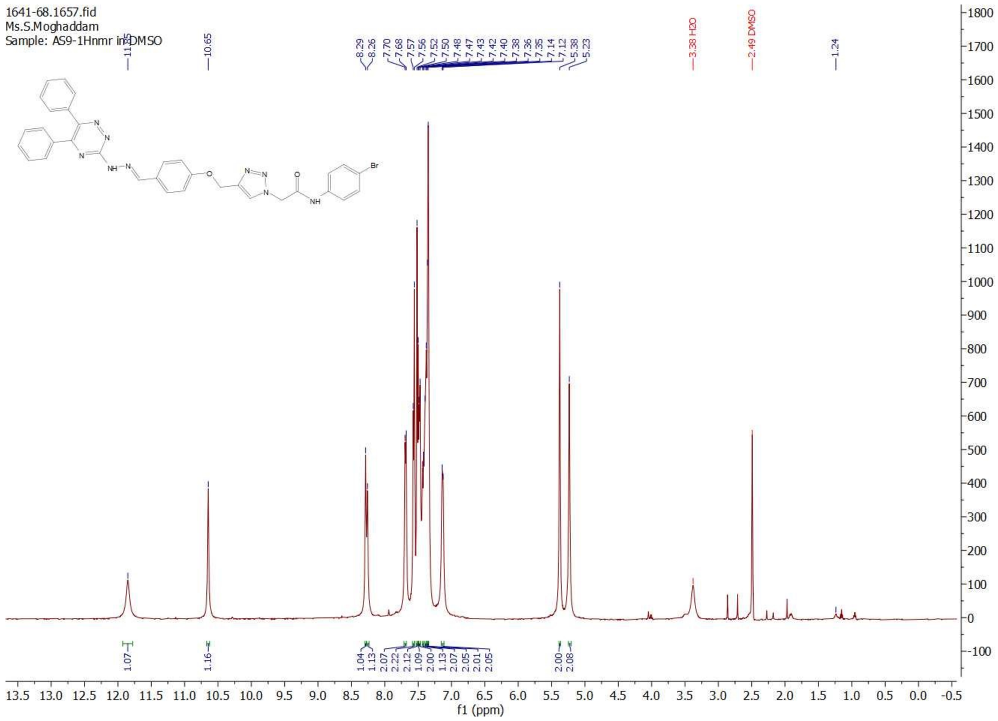

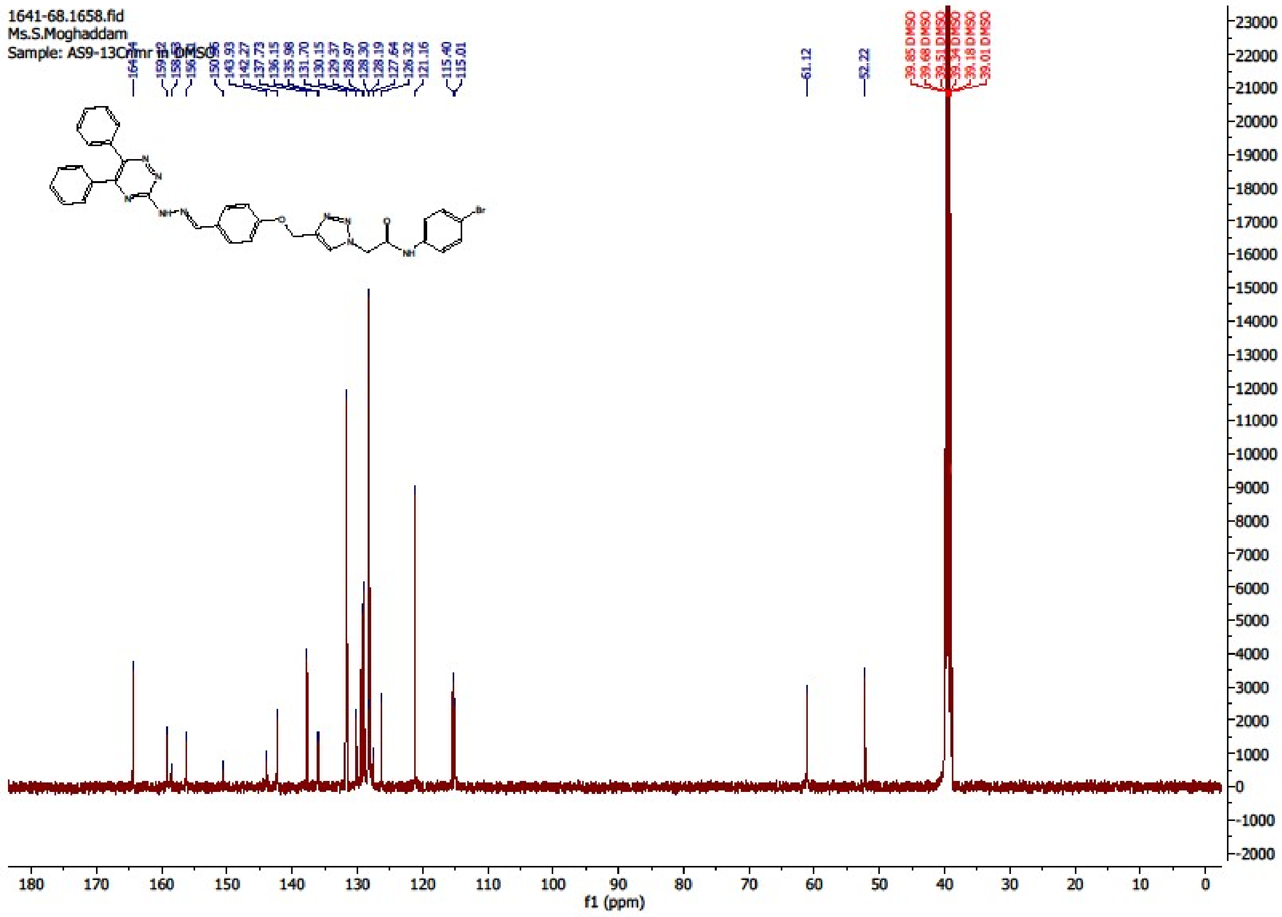

(E)-2-(4-((4-((2-(5,6-diphenyl-1,2,4-triazin-3-yl)hydrazineylidene)methyl)phenoxy)methyl)-1H-1,2,3-triazol-1-yl)-N-(2-nitrophenyl)acetamide (**13l**)

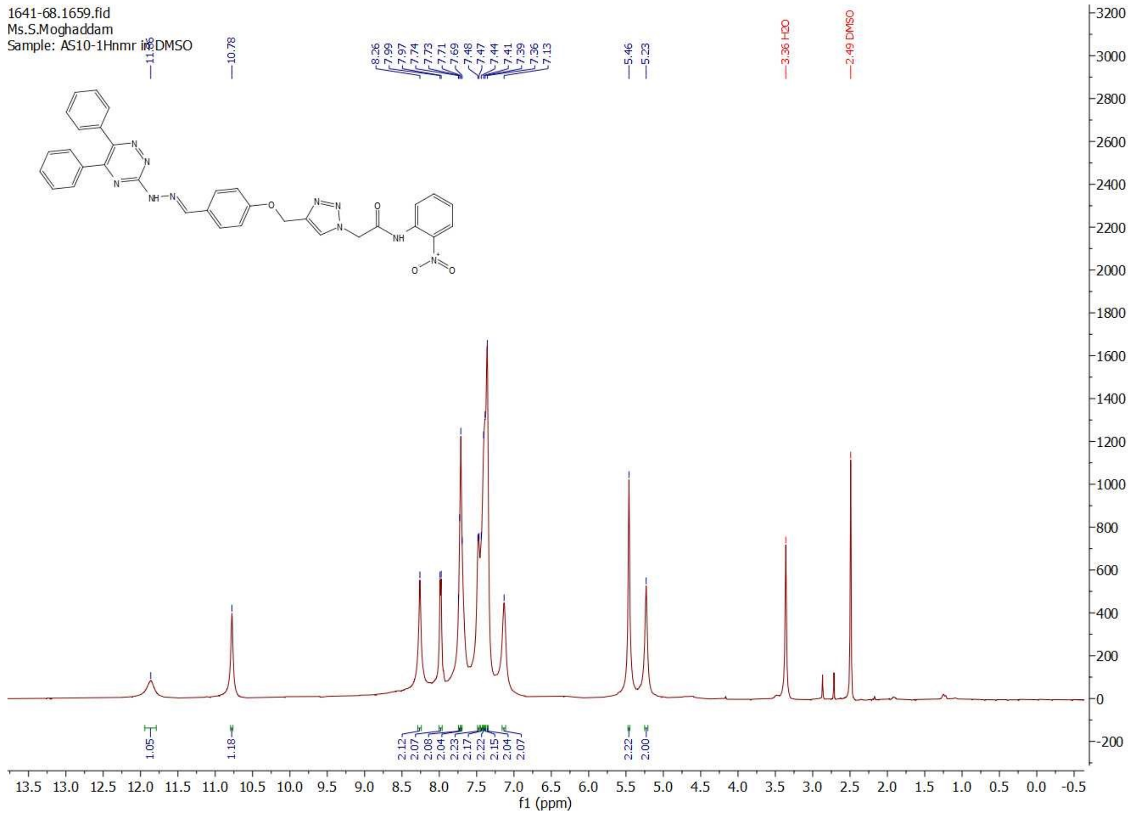

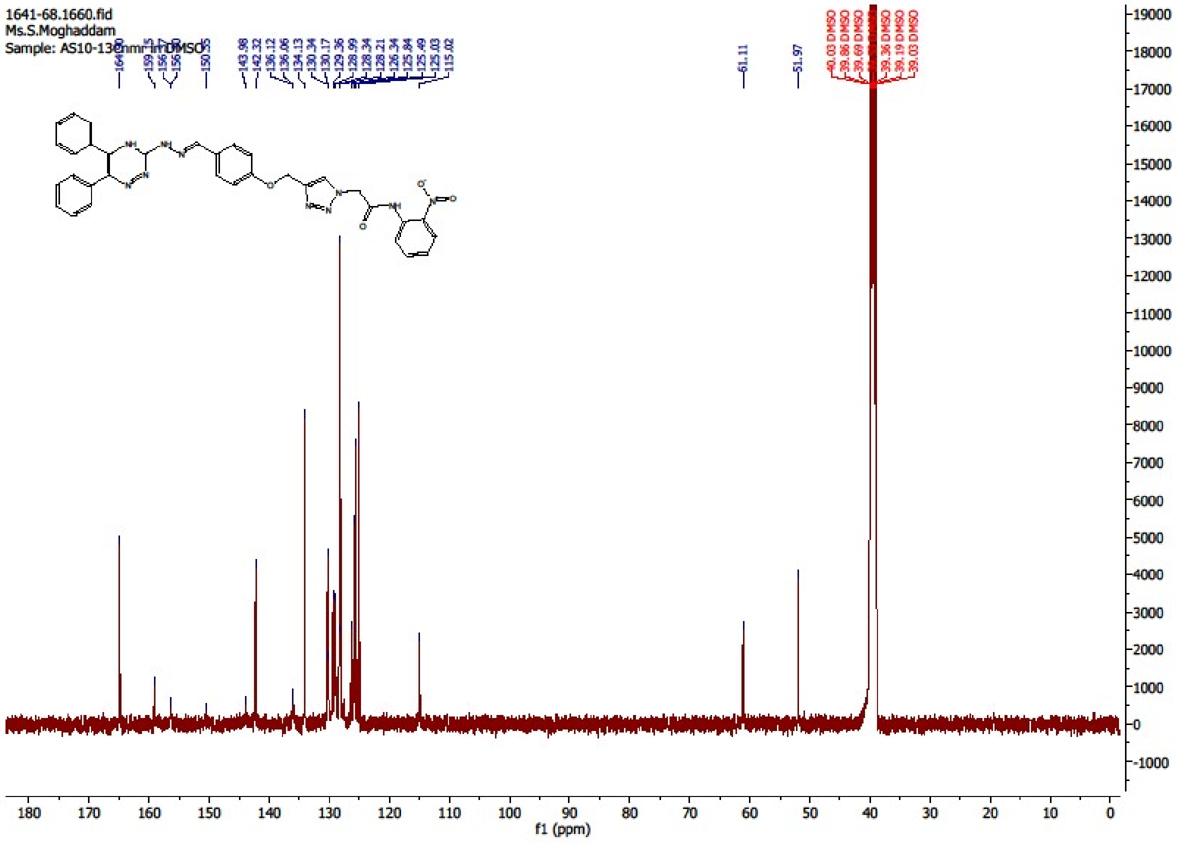

(E)-N-benzyl-2-(4-((4-((2-(5,6-diphenyl-1,2,4-triazin-3-yl)hydrazineylidene)methyl)phenoxy)methyl)-1H-1,2,3-triazol-1-yl)acetamide (**13m**)

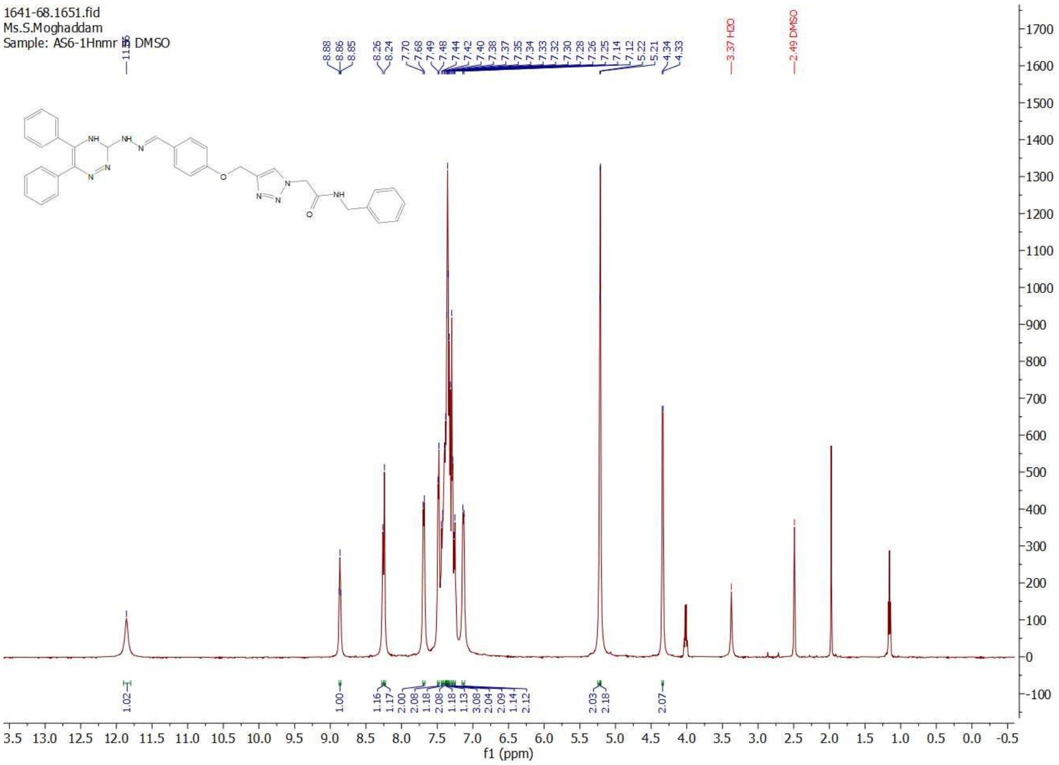

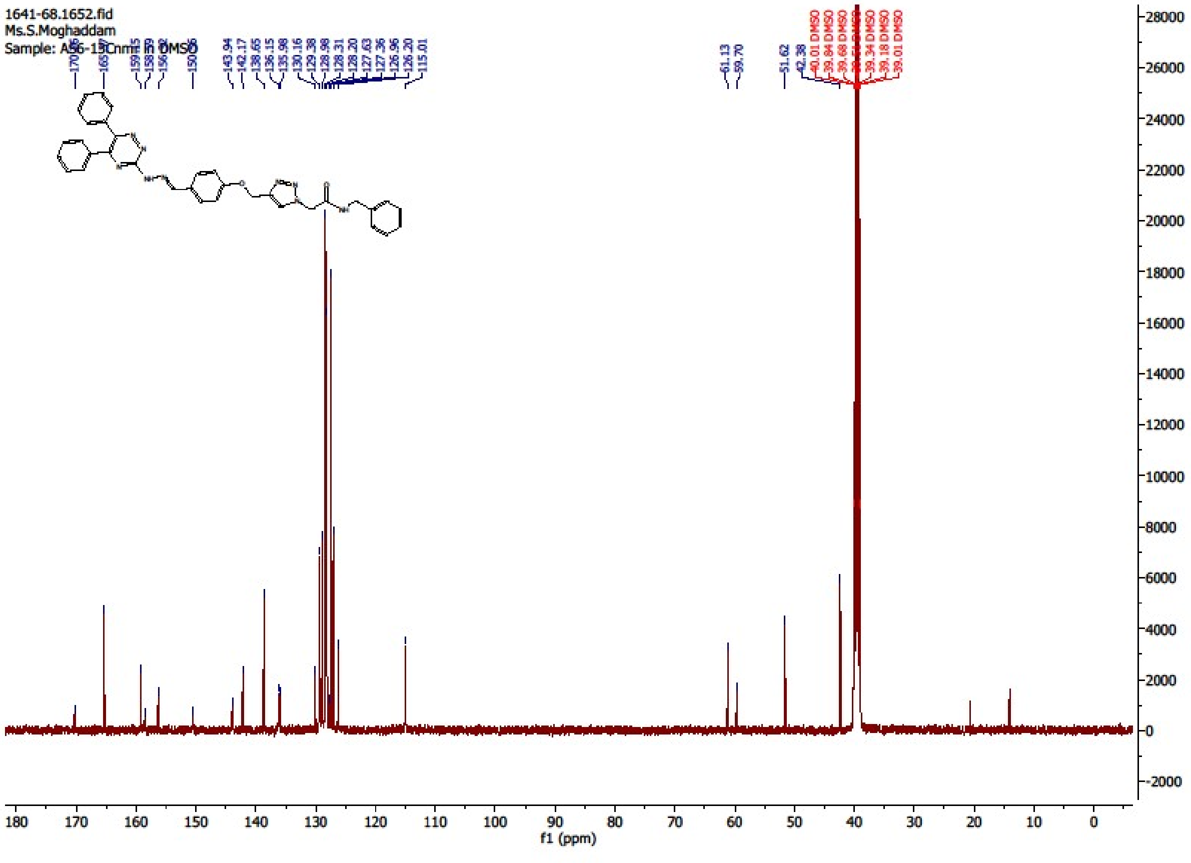

(E)-2-(4-((4-((2-(5,6-diphenyl-1,2,4-triazin-3-yl)hydrazineylidene)methyl)phenoxy)methyl)-1H-1,2,3-triazol-1-yl)-N-phenethylacetamide (**13n**)

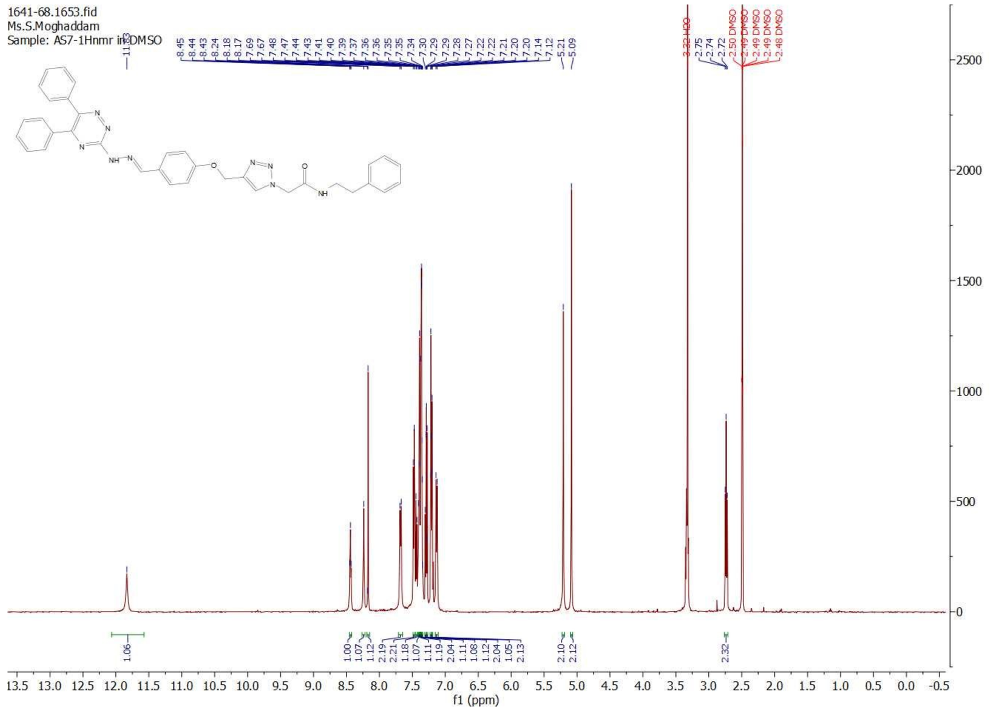

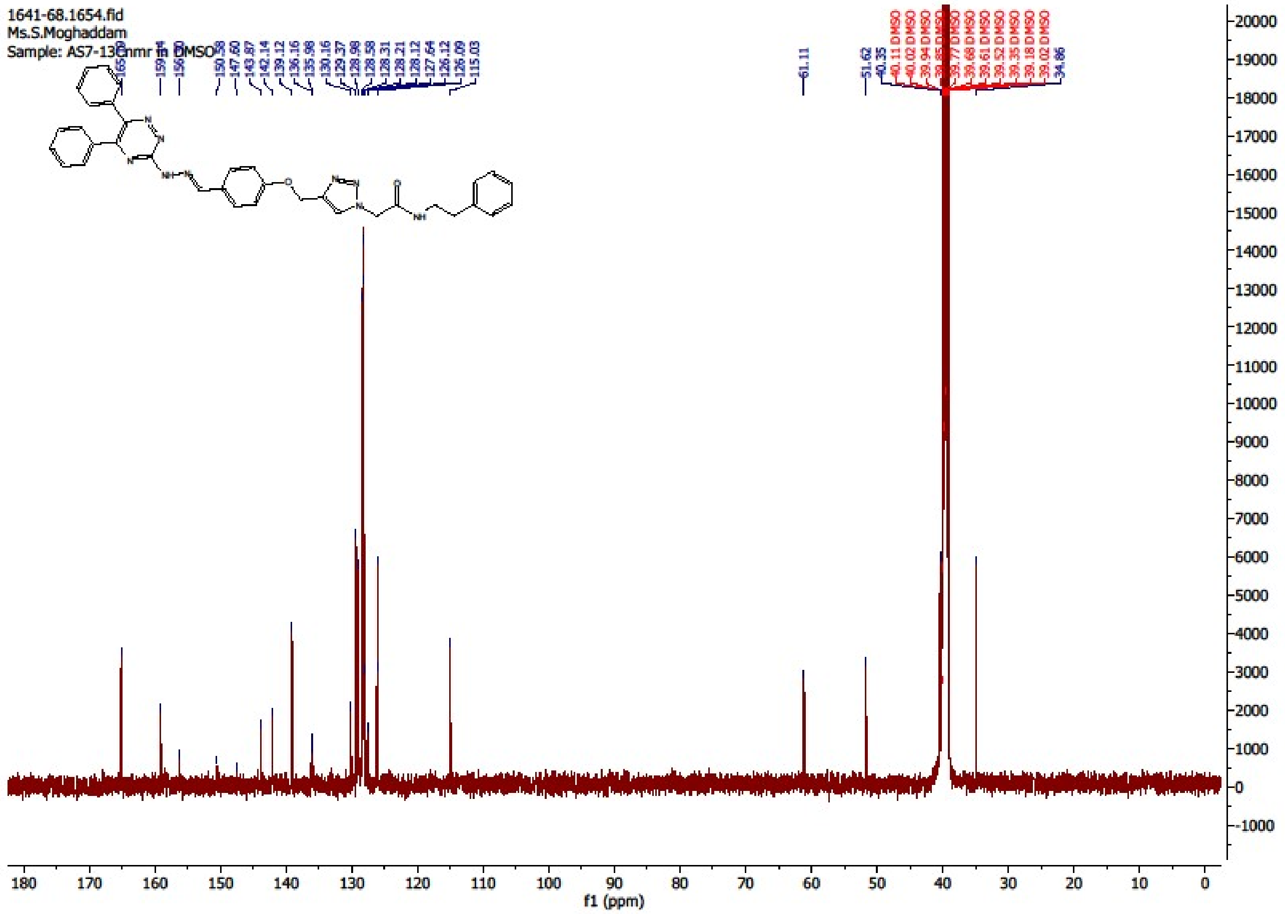

The inhibition percentage of the synthesized compounds **13a-n** against α-glucosidase.

Compound	In (%)
13a	95
13b	98
13c	100
13d	75
13e	100
13f	100
13g	79
13h	87
13i	100
13j	83
13k	69
13l	69
13m	90
13n	100
Acarbose	50

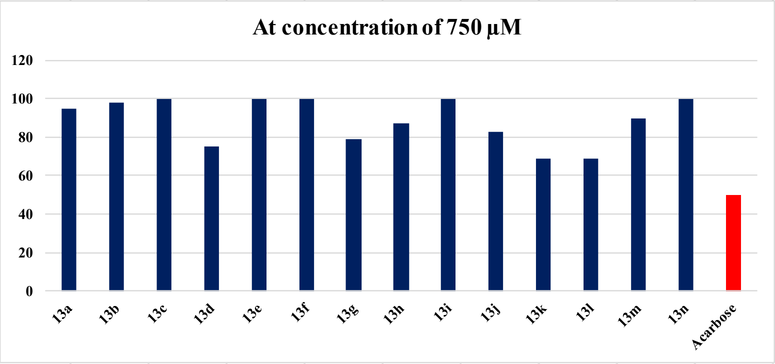

IC$_{50}$ determination curves for the synthesized compounds.

13f

Conc.	OD Blank	OD Total	OD Final	Inhibation
120	0.09	0.19	0.1	100
90	0.067	0.297	0.23	98
60	0.023	0.363	0.34	89
30	0.097	0.507	0.41	72.66666667
15	0.012	0.672	0.66	56
			IC50	10.90801366

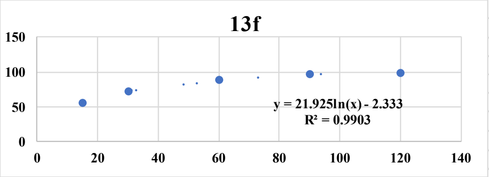

13i

Conc.	OD Blank	OD Total	OD Final	Inhibation
120	0.08	0.2	0.12	100
90	0.045	0.315	0.27	98
60	0.012	0.302	0.29	84
30	0.067	0.446	0.379	74.73333333
15	0.034	0.733	0.699	53.4
			IC50	11.72687725

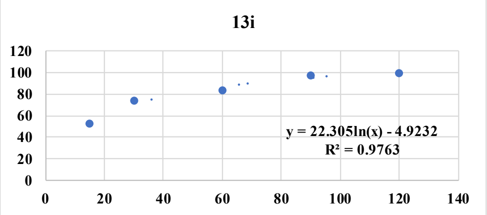

13b

Conc.	OD Blank	OD Total	OD Final	Inhibation
120	0.09	0.19	0.1	98
90	0.067	0.287	0.22	93
60	0.109	0.379	0.27	83
30	0.104	0.599	0.495	67
15	0.034	0.711	0.677	54.86666667
			IC50	12.50116195

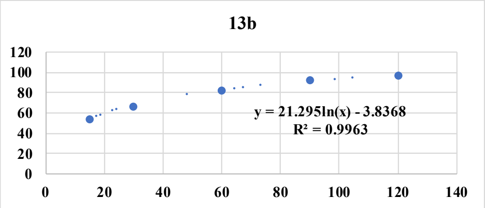

13e

Conc.	OD Blank	OD Total	OD Final	Inhibation
120	0.08	0.18	0.1	100
90	0.089	0.199	0.11	100
60	0.05	0.28	0.23	95
30	0.1	0.356	0.256	82.93333333
15	0.067	0.523	0.456	69.6
			IC50	3.732183548

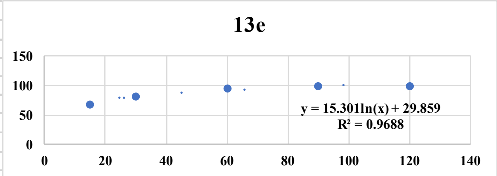

13a

Conc.	OD Blank	OD Total	OD Final	Inhibation
120	0.022	0.252	0.23	94
90	0.032	0.272	0.24	93
60	0.078	0.328	0.25	89
30	0.045	0.325	0.28	81.33333333
15	0.102	0.454	0.352	76.53333333
			IC50	0.807765774

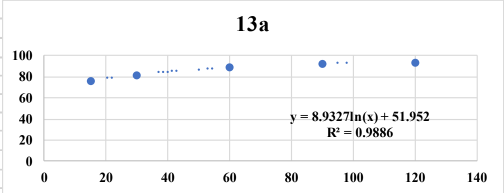

13m

Conc.	OD Blank	OD Total	OD Final	Inhibation
120	0.056	0.236	0.18	89
90	0.034	0.224	0.19	88.3
60	0.102	0.302	0.2	87
30	0.023	0.237	0.214	80
15	0.054	0.297	0.243	70
			IC50	0.900087626

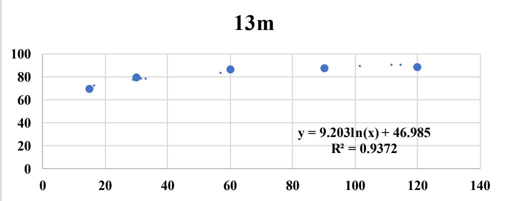

13n

Conc.	OD Blank	OD Total	OD Final	Inhibation
120	0.089	0.259	0.17	98
90	0.034	0.214	0.18	93
60	0.0212	0.2312	0.21	90
30	0.034	0.264	0.23	84.66666667
15	0.045	0.356	0.311	79.26666667
			IC50	0.511407661

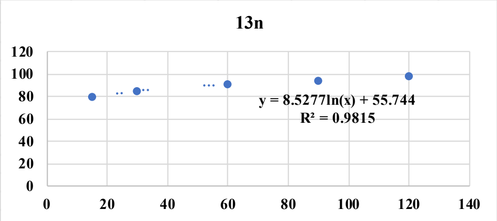

13c

Conc.	OD Blank	OD Total	OD Final	Inhibation
120	0.023	0.193	0.17	97
90	0.056	0.246	0.19	96
60	0.023	0.263	0.24	93
30	0.102	0.392	0.29	80.66666667
15	0.093	0.471	0.378	74.8
			IC50	1.828412814

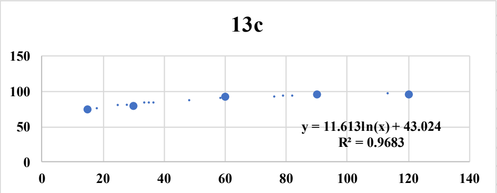

13k				
Conc.	OD Blank	OD Total	OD Final	Inhibation
120	0.023	0.193	0.17	67
90	0.012	0.432	0.42	65
60	0.098	0.528	0.43	60
30	0.07	0.579	0.509	56.12068966
15	0.065	0.646	0.581	49.9137931
			IC50	15.30829842

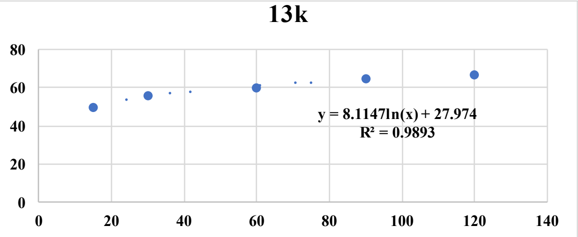

13l				
Conc.	OD Blank	OD Total	OD Final	Inhibation
120	0.043	0.423	0.38	67
90	0.098	0.488	0.39	65
60	0.023	0.493	0.47	59
30	0.056	0.654	0.598	48.44827586
15	0.067	0.789	0.722	37.75862069
			IC50	34.23790396

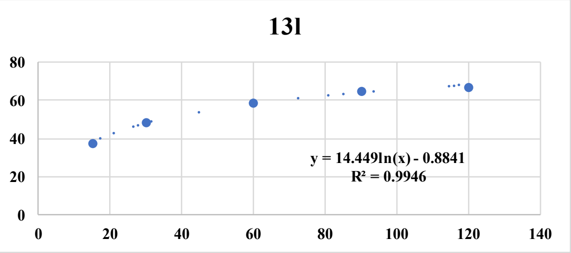

13d				
Conc.	OD Blank	OD Total	OD Final	Inhibation
120	0.067	0.407	0.34	74
90	0.078	0.448	0.37	72
60	0.034	0.424	0.39	68
30	0.039	0.443	0.404	65.17241379
15	0.109	0.56	0.451	61.12068966
			IC50	2.548487383

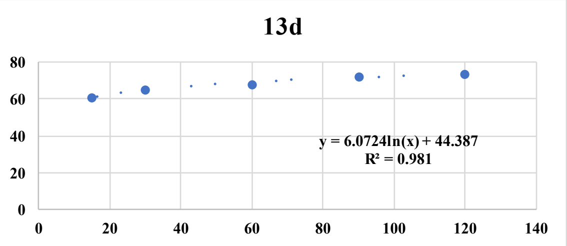

13h				
Conc.	OD Blank	OD Total	OD Final	Inhibation
120	0.103	0.303	0.2	87
90	0.098	0.318	0.22	86
60	0.02	0.25	0.23	83
30	0.102	0.342	0.24	79.31034483
15	0.12	0.406	0.286	75.34482759
			IC50	0.193334519

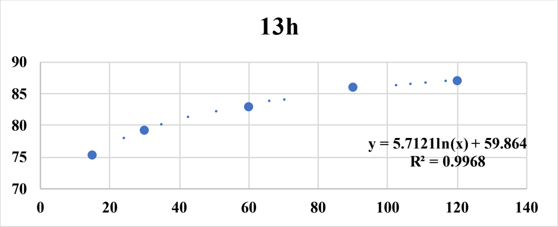

13j				
Conc.	OD Blank	OD Total	OD Final	Inhibation
120	0.023	0.253	0.23	83
90	0.012	0.262	0.25	81
60	0.109	0.369	0.26	79
30	0.14	0.425	0.285	75.43103448
15	0.103	0.416	0.313	73.01724138
			IC50	0.122717251

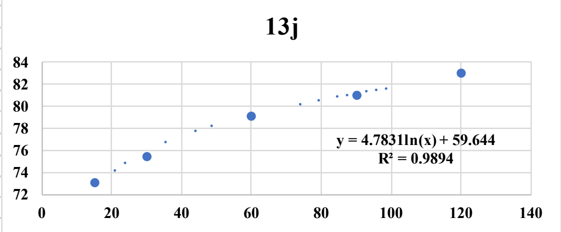

13g				
Conc.	OD Blank	OD Total	OD Final	Inhibation
120	0.034	0.154	0.12	79
90	0.038	0.278	0.24	78
60	0.098	0.398	0.3	76
30	0.103	0.425	0.322	72.24137931
15	0.034	0.393	0.359	69.05172414
			IC50	0.312464222

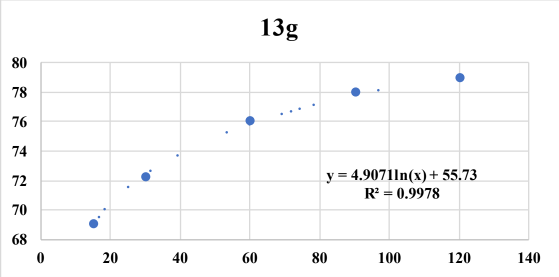

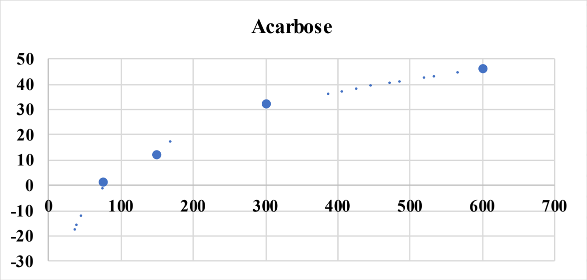

Acarbose				
Conc.	OD Blank	OD Total	OD Final	In (%)
600	0.023	0.573	0.55	46
300	0.034	0.684	0.65	32
150	0.103	0.986	0.883	12
75	0.101	1.251	1.15	1
32.5	0.098	1.398	1.3	
			IC50	750

Kinetic study on compound **13j**

1/S	1/V0	1/V1	1/V2	1/V3	1/V4
0.25	26	46	66	84	106.744
0.333333	32	52	72	92.38208	109.17
0.5	36	56	76	96.45776	121.3
1	60	85	107	127.68	145.56
2	100	134	156	176.6128	200

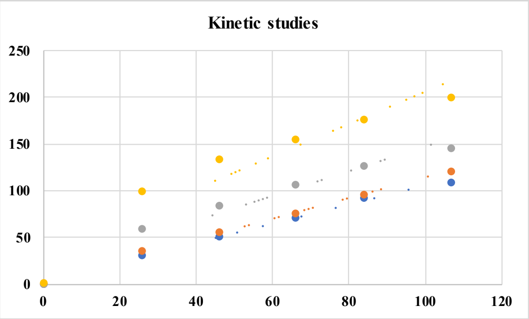

Supplement: RA-015-D5RA06909B-s001 [file RA-015-D5RA06909B-s001.pdf]
